# Characteristics and outcomes of pharmacy-supported transitions of care interventions in emergency departments: a scoping review

**DOI:** 10.1007/s11096-025-02057-0

**Published:** 2025-12-04

**Authors:** Eman Alhmoud, Waad Elamin, Raja Barazi, Zeana Alkudsi, Farah Zahrah, Muhammad Abdul Hadi

**Affiliations:** 1 QU Health Sector, Qatar University, Doha, Qatar; 2 Pharmacy Department, Hamad Medical Corporation, Doha, Qatar; 3 Department of Clinical Pharmacy and Practice, College of Pharmacy, QU Health Sector, Qatar University, P.O. Box 2713, Doha, Qatar

**Keywords:** Emergency department, Health services research, Pharmacists, Pharmaceutical services, Transitional care

## Abstract

**Introduction:**

Transitions of care (ToC) services are essential for maintaining care continuity. The complex and fast-paced nature of care and high patient turnover in emergency departments (EDs) create unique challenges and opportunities for improving transitional care. Although the benefits of pharmacy-supported ToC interventions are established in non-ED settings, there is a lack of evidence exploring their characteristics and outcomes in EDs.

**Aim:**

We aimed to identify and present the available evidence regarding the characteristics and outcomes of pharmacy-supported ToC interventions beyond medication reconciliation, as the sole intervention, in EDs.

**Method:**

This review was conducted in accordance with the Joanna Briggs Institute methodology and reported following the Preferred Reporting Items for Systematic Reviews and Meta-Analysis Extension for Scoping Reviews guidelines. A literature search was performed across PubMed, Embase, CINAHL, Web of Science, and grey literature from their inception until 22/12/24. The search included terms related to pharmacy, transitional care, and EDs. Data was extracted using a custom tool adapted from the Template for Intervention Description and Replication checklist, which was used to assess the articles’ compliance with the items.

**Results:**

A total of 64 publications were included. Most studies (n = 58) enrolled adult patients, with 13 focusing on older adults. Most interventions were delivered by pharmacists in collaboration with other healthcare providers in 64% of studies. Interventions were most implemented post-discharge (54.7%), followed by arrival to the ED (42.2%). Around 90.6% of interventions included two or more activities, combining medication reconciliation, discharge planning, and follow-up care. Most studies focused on health utilization metrics (e.g., readmission rates) as their outcomes (28.8%). Positive effects were observed on medication safety, antibiotic stewardship, patient satisfaction, and resource use. However, pediatric populations and intrahospital transitions were underrepresented.

**Conclusion:**

This scoping review highlights the potential of pharmacist-supported transitional care interventions within EDs. The role of pharmacists in ToC interventions in emergency settings is evidently growing. Despite this, critical gaps persist in reporting and implementing these interventions. Future research is needed to systematically explore such initiatives and evaluate their implementation and long-term impact.

**Supplementary Information:**

The online version contains supplementary material available at 10.1007/s11096-025-02057-0.

## Impact statements

- Poor and inconsistent reporting of intervention characteristics, fidelity, tailoring, and provider characteristics remain critical barriers to the replication, evaluation, and implementation of pharmacy-supported transition of care (ToC) interventions in emergency departments (EDs) across diverse settings.
- Although patients are highly vulnerable to medication errors during within-hospital transitions, this touchpoint remains underrepresented in the current landscape of pharmacy-supported ToC within EDs.
- Institutional support and multidisciplinary collaboration are essential for successful ED transitional care programmes.
- Future practice must expand focus to vulnerable and underrepresented populations (e.g., pediatrics and older adults) who are most likely to benefit from pharmacy-supported TOC interventions in EDs.

## Introduction

Transitions of care (ToC) are movements of patients between healthcare settings, levels of care, or providers [1]. These transition “touchpoints” are vulnerable to medication errors and adverse drug events (ADEs). ToC services are therefore critical to continuity. Approximately half of adults discharged from hospitals experience a medication discrepancy, with around 20% encountering an ADE [2, 3].

Emergency departments (EDs) present unique challenges and opportunities for ToC due to the acute and complex nature of care. Reported barriers include high patient turnover, inconsistent referral systems, time-sensitive care, miscommunication, and limited awareness of community-based services [4]. Patients often encounter unfamiliar health providers in the ED, complicating follow-up care [5].

The safety implications of these challenges are well documented [6–9]. A retrospective study found that 16.5% of ED discharge prescriptions contained errors, with error rates exceeding 50% for antidiabetics and antiplatelets [6]. Similarly, non-adherence to at least one medication was observed in over 40% of ED-discharged patients [7], further increasing the risk of treatment failure and ADEs.

In response, professional pharmacy and healthcare bodies endorsed emergency medicine pharmacists' roles in facilitating ToC [10–13]. Those guidelines urge institutional support for pharmacist-supported initiatives to reduce return visits and readmissions [10–13]. Although pharmacists' roles in transitional care services in non-ED settings are well recognized [14, 15], their involvement in emergency settings remains largely unexplored.

Current evidence is mostly limited to medication reconciliation as a standalone intervention, with less attention directed to broader interventions.

Given the complexity of ED care and the potential for pharmacist-supported services to reduce ToC-related risks, it is important to explore the scope and characteristics of pharmacy-supported interventions in EDs.

### Aim

This scoping review aimed to identify, map, and describe the available literature on pharmacy-supported ToC interventions in EDs, beyond medication reconciliation. Specifically, it explored study designs, intervention types, providers/recipients, materials and procedures, delivery mode/timing, tailoring, fidelity measures, and outcomes. It also assessed the extent to which these characteristics were reported against the Template of Intervention Description and Replication (TIDieR) framework.

## Method

The scoping review was conducted in accordance with the JBI methodology for scoping reviews (JBI) and reported following the Preferred Reporting Items for Systematic Reviews and Meta-Analysis extension for Scoping Reviews (PRISMA-ScR) guidelines [16, 17]. An a priori protocol [18] detailing the study’s methodology is registered in The Open Science Framework (OSF) [19] (10.17605/OSF.IO/U5FT7).

### Search strategy

MEDLINE (via PubMed), Embase, CINAHL, and Web of Science were searched from inception until 22/12/2024 was performed. This was supplemented by a grey literature search in Google Scholar and a manual bibliography screening. No language restrictions were applied.

The search consisted of database-controlled vocabulary where available and free-text keywords related to three concepts: pharmacists, transitional care, and emergency departments. Boolean operators (OR, AND) and truncations (*) were utilized as necessary. The strategy was tailored to each database. (Appendix Tables S1-S3).

### Article selection

**Fig. 1:**
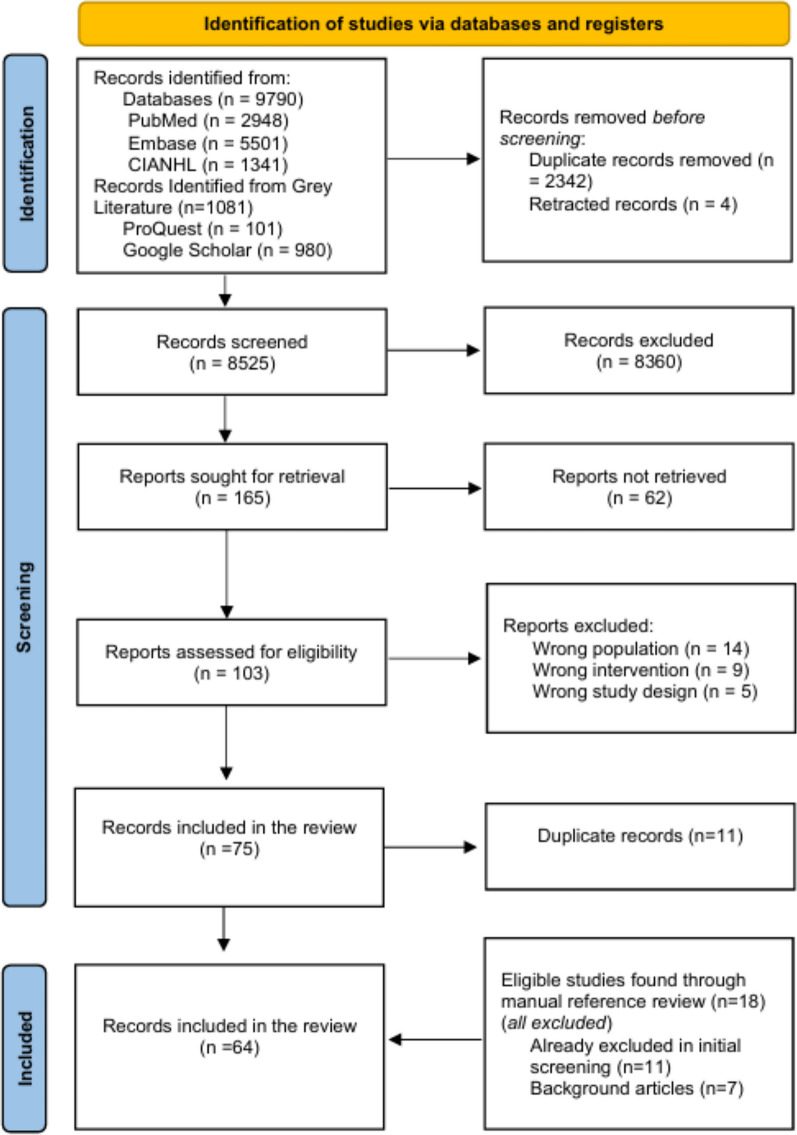
PRISMA flow chart showing the inclusion process

All identified citations were uploaded into EndNote 20, updated, de-duplicated, and then imported into Rayyan® (https://rayyan.ai) [20] to track the screening process. Title and abstracts were independently assessed by three reviewers (EN, FA, RB) against inclusion/exclusion criteria (Table 1). Studies reporting medication reconciliation as their sole intervention were excluded, as evidence surrounding the topic is well established, including two systematic reviews and meta-analyses evaluating it [14, 15]. Therefore, we focused on other interventions either alone or combined with medication reconciliation. Full text screening was completed independently by four researchers (EN, ZA, RB, WE). Discrepancies between two reviewers were resolved through discussion and, when necessary, by consulting a third reviewer. Reference lists and bibliographies of included studies were manually assessed for eligibility. Reasons for exclusion are in Fig. 1.

**Table 1:** The inclusion and exclusion criteria used to assess the eligibility of articles

Inclusion criteria	Exclusion criteria
**Intervention personnel**: Pharmacists, pharmacy students, pharmacy technicians, or pharmacy interns working individually or collaboratively as part of a multidisciplinary team	**Qualitative-only studies:** studies that rely exclusively on qualitative data (e.g. Interviews, focus groups) without reporting any quantitative outcome measures
**Patient population:** Patient admitted to, transferred within, or discharged from an emergency department setting, including emergency short-stay units (SSUs)	**Medication reconciliation as sole intervention or no ToC element:** Studies where medication reconciliation is the sole intervention without additional ToC processes or reporting pharmacist-led activities unrelated to transitional care points (e.g. General medication safety audit or routine medication verification)
**Transition point:** Description of interventions linked to at least one transitional care point, such as: Arrival to the emergency department Transfer within the facility Transfer to another healthcare facility Discharge from the emergency department Post-discharge follow-up	**Non-ED settings:** Studies reporting ToC interventions in primary care, outpatient clinics, or long-term care facilities without an ED component
**Intervention scope:** Eligible interventions may involve one or more pharmacy-supported activities, such as: Medication reconciliation (as part of a broader intervention package) Comprehensive Medication Management (CMM) Discharge planning/ timely communication of information and care-coordination Post-discharge follow-up Medication and self-management education Medication acquisition assistance	**Non-eligible publication types:** Reviews, commentaries, editorials, letters to the editor, and study protocols will not be included

### Data extraction and synthesis

Four investigators (EN, ZN, RB, WE) extracted data in pairs using a custom tool (Tables S4, S5) adapted from the TIDieR checklist [21]. The extracted data included study and intervention characteristics (description of intervention and control if applicable, transition points, intervention intensity, outcomes, overall effect, and TIDieR items). In cases where additional information was required, authors were contacted via email.

Intervention intensity was defined as the number of distinct pharmacy-supported transitional care activities in each intervention package. Activities included: medication reconciliation (when part of a broader package), comprehensive medication management (CMM), discharge planning/care coordination, post-discharge follow-up, medication education, and medication acquisition assistance.

The TIDieR checklist consists of 12 items:

1. **Brief Name**: *Title or label of the intervention*
2. **Why**: *Rationale or underlying theory*
3. **What (Materials)**: *Physical/informational resources used*
4. **What (Procedures)**: *Core activities and procedures*
5. **Who Provided**: *Qualifications and background of providers*
6. **How**: *Mode and format of delivery*
7. **Where**: *Intervention setting characteristics*
8. **When and How Much**: *Frequency and duration*
9. **Tailoring**: *Any planned customization*
10. **Modifications**: *Changes made during implementation*
11. **How Well (Planned Fidelity)**: *Adherence strategies*
12. **How Well (Actual Fidelity):** *Intervention implementation accuracy*

Additionally, outcome measures were categorized using the American College of Clinical Pharmacy (ACCP) framework proposed by King et al. [22] which groups them into process, intermediate outcomes, and final outcome measures.

- **Process outcomes**: healthcare activities, such as identifying medication discrepancies (QM-1) or therapy problems (QM-2)
- **Intermediate outcome measures**: measures that influence quality related to major outcomes, such as the resolution of discrepancies (iQM-1) and therapy problems (iQM-2)
- **Final outcome measures**: impact of healthcare services, such as the measures of morbidity or mortality. Those are categorized into Health Care Utilization (QM-3), Patient Satisfaction and Engagement (QM-4), and Economics (QM-5).

Extracted study characteristics were quantified, summarized, and categorized using descriptive analysis.

## Results

The search retrieved 9790 articles. After removing duplicate and retracted records, 8525 were screened by title and abstract (Fig. 1). This yielded 165 full-text articles assessed for eligibility, resulting in the final inclusion of 64 publications [23–86].

### Characteristics of the studies

Table 2 presents the included studies’ characteristics. Sample sizes ranged from 3 to 11,194, encompassing both small-scale exploratory studies and large-scale investigations. Study designs were most commonly quasi-experimental (n = 19), followed by randomized controlled trials (RCTs) (n = 9). The remaining studies included various observational designs, case series, and quality improvement initiatives. The studies originated from 10 countries, with the majority conducted in the United States (n = 41, 64.1%), followed by Australia (n = 13, 20.3%) and Europe (n = 6, 9.4%). The majority (n = 58) were single-center, with only six adopting a multicenter approach. Most focused on patients discharged directly from EDs. The duration of intervention delivery varied markedly, ranging from 3 weeks to 36 months. Fifty-eight studies enrolled adults, with 13 (20%) focusing on older adults. Nineteen (n = 30%) targeted patients discharged with pending culture or laboratory results.

**Table 2:** Overall characteristics of the included studies, n = 64

First author, Publication year	Country	Study design	Study follow-up period (days)	Inclusion criteria (targeted population)	General description of intervention	Intervention intensity^a^	Primary outcome descriptions	Overall effect of the intervention
Kaucher, 2025 [23]	USA	Retrospective, pre-post implementation analysis	90 days	Patients presenting to the ED requiring medical treatment for sexual assault, who received a 3-day starter pack or a 28-day complete nPEP regimen	Counselling by Emergency Medicine clinical pharmacists and a complete 28-day supply of nPEP free of charge to the patient at ED discharge	2	Percentage of patients who had at least one follow-up HIV screening within 6 months after initial SANE evaluation in the ED Rate of compliance with at least one HIV screening Total number of screening occurrences related to 1-, 3-, and 6-month follow-up recommendations	Favors intervention
Boot, 2024 [24]	USA	Retrospective, quality improvement project	30 days post-discharge	Adults discharged from the ED with a subsequent positive microbial culture result for suspected urinary, sexually transmitted, or bloodstream infections	A clinical pharmacist reviewed daily culture reports, assessed diagnosis, culture, allergies, etc., and recommended or initiated antimicrobial therapy, contacted patients, phoned prescriptions, and documented in EHR	2	Percentage of patients receiving optimal therapy after the culture callback was completed	Favors intervention
Reilly, 2024 [25]	Australia	Descriptive analysis	28 days post-discharge	Older adults (70 years or older), discharged from ED, ≥ 3 comorbidities, polypharmacy (≥ 5 medications daily)	GEDI pharmacist-led review using STOPP/START criteria, medication reconciliation, patient education, updated medication list, and written/electronic recommendations sent to physician	3	Medication changes based on STOPP/START criteria GP acceptance of recommendations Patient satisfaction	Potential benefit
Lee, 2024 [26]	Australia	Prospective pre-post intervention study	180 days	Adults discharged from the Short Stay Unit with a prescription, regardless of their diagnosis and discharge destinations	EM pharmacists attended ward rounds, participated in medication management decision-making making and planned discharge prescriptions	3	Proportion of discharge prescriptions that met all five rights (5Rs)	Favors intervention
Selman, 2024 [27]	USA	Observational prospective cohort	365 days	Older adults (age 65 years or older), presented to ED after a fall	Pharmacist review of medications for older patients presenting to the ED after falls	4	Return visit for fall	No difference
Martínez, 2024 [28]	Chile	Randomized, controlled pragmatic trial	30 days	Adults admitted to ED for non-traumatologic causes, between 9 am and 11 pm, Monday to Friday, with triage categories C2 (unstable patient with evident emergency), C3 (medium risk with evident emergency), and C4 (mild urgency)	Clinical pharmacist who reviewed pharmacotherapy, made recommendations to the medical team, and provided education to patients and their caregivers on the therapeutic plan	3	Reconsultations to the same health center within 30 days after discharge because of the same complaint	Favors intervention
Maleki, 2024 [29]	Australia	Retrospective observational study	NA	Adults admitted to the General Medicine Unit via ED, identified as ‘High-Needs’ for pharmacy services using a previously validated tool that screened patients at higher risk of medication-related adverse outcomes	ED pharmacists would complete the BPMH for High Needs Patients	2	Rate of medication errors	Favors intervention
Sofeso, 2024 [30]	USA	Quality improvement project	NA	Adults discharged from ED with an electronic prescription for UTI treatment with select antibiotics	EMP prospectively reviewed orders based on adherence to local guidelines, consulted with the prescribing clinician to provide recommendations to address any medication-related issues identified, and verified orders	2	Percentage of medication errors (defined as a composite of appropriate antibiotic agent, dose, frequency, and treatment duration)	Potential benefit
Tran-Nguyen, 2024 [31]	Australia	Retrospective cohort study	28 days	Older adults taking ≥ 3 medications, and presenting ED with falls, cognition changes, or reduced mobility, and planned for discharge home	A pharmacist provided comprehensive medication management consultations, discharge liaison services, and other pharmacy-related interventions	3	Unplanned hospital readmissions	Favors intervention
Atey, 2024 [32]	Australia	Retrospective concurrent, controlled, pragmatic trial	Until the initial inpatient charting	Adults presenting to the ED with regular medication prior to admission, and who were admitted to the general medicine unit, emergency medicine unit, or mental health units	PPMC pharmacist documented BPMH, collaborated with medical officers to co-develop treatment plans, and chart medications in the ED	3	The duration, in hours, from ED presentation to time-critical medicines first dose administration Completeness of initial medication orders Conduct of VTE risk assessments	Favors intervention
Wang, 2024 [33]	USA	Observational, pre-post intervention study	28 days	Adults discharged from ED with an antibiotic and a subsequent negative culture result for urine and sexually transmitted infection	Expanded culture callback protocol for deprescribing	1	Antibiotic-free days	Potential benefit
Kofoed, 2023 [34]	USA	Quality improvement project	30 days	Adults diagnosed with an infectious disease of the genitourinary system, cystitis, or dysuria, discharged from the ED with an antibiotic and subsequent negative urine culture	Pharmacist-driven follow-up results for negative urine culture	1	Proportion of patients contacted to stop antibiotics prescribed for urinary symptoms when the urine culture was negative	Favors Intervention
Pham, 2023 [35]	USA	Retrospective study	30 days	Adults discharged from ED with a urine culture collected in ED	ED pharmacists were privileged to independently adjust antibiotic regimens based on urine culture results within the specifications of the protocol	2	Time to intervention after culture results were released (time from the culture result to when a progress note was charted regarding the result)	Neutral
Jovevski, 2023 [36]	USA	Retrospective quasi-experimental before-after study	60 days	Older adults (age 75 years or older), with a positive screening tool used to identify patients at risk of poor outcomes after their ED visit, discharged home from ED, had a primary care provider, and a pharmacy medication review performed and documented in the EHR	Standardized process to screen for high-risk older adults	4	PIM Deprescribing events	Favors intervention
Andrade, 2023 [37]	USA	Retrospective, quasi-experimental pre-post study	30 days	Adults with confirmed positive microbial culture of ESBL, MRSA, and VRE at any site, and discharged from the ED	EMPs review positive MDR microbiology culture results for ED-discharged patients and determine treatment appropriateness, adjust antimicrobials as and communicate with patients as needed without physician notification	1	ED revisit rates within 30 days due to antimicrobial treatment failure, defined as lack of resolution or worsening of infection	Favors intervention
Dunn, 2023 [38]	USA	Randomized Controlled Trial	30 days	Adults who have a primary care provider, were discharged home from ED, are taking at least 8 different medications at ED admission (inclusive of as-needed medications), and speak and understand English	Medication reconciliation after ED discharge and comparing to what the patient was taking at home	2	Unplanned hospital utilization within 30 days of ED discharge (a composite of unexpected ED visits and/or hospital admissions)	Neutral
Atey, 2023 [39]	Australia	Pragmatic, parallel controlled study	NA	Older people (65 years or older), presenting to the ED with ≥ 1 regular medication, subsequently admitted to AMU, and receiving their first medication reconciliation on the ward within 48 h after transferring from ED	PPMC pharmacist documented BPMH, collaborated with medical officers to co-develop treatment plans, and chart medications in ED	3	Percentage of patients prescribed at least one PIM on ED departure	Favors intervention
Atey, 2023 [40]	Australia	Pragmatic, parallel controlled study	NA	Adults presenting to the ED with ≥ 1 medication, subsequently admitted to a general medicine unit (GMU) or emergency medicine unit (EMU)	PPMC pharmacist documented BPMH, collaborated with medical officers to co-develop treatment plans, and chart medications in ED	3	Length of stay (LOS: time from ED presentation to discharge (in days), Relative stay index (RSI)	Mixed results
Atey, 2023 [41]	Australia	Controlled concurrent pragmatic evaluation	Within 2 days of transfer from ED	Older adults (65 years or older), taking any prescribed preadmission medication, or having a mental health admission, questionable adherence, or any medication concerns, using a patient risk assessment framework, intended to be admitted through ED to an AMU	PPMC pharmacist documented BPMH, collaborated with medical officers to co-develop treatment plans, and chart medications in ED	3	Proportion of patients who had at least one medication error Proportion of patients who had at least one medication discrepancy	Favors intervention
Zhao, 2023 [42]	USA	Retrospective pre-post study	30 days	Patient discharged from ED with urine culture collected in ED, not finalized at time of ED discharge	Pharmacists review positive urine cultures post-ED discharge to provide treatment recommendations	3	Receipt of guideline-appropriate antimicrobial management Acceptance rate of pharmacist recommendations	Favors Intervention
Benson, 2023 [43]	USA	Quality improvement study	3 months	Patients discharged from ED, with a urine culture collected in ED, regardless of age group or culture result	The pharmacist conducted a patient chart review for each urine culture and determined whether an intervention or modification was required	2	Number of discontinued antibiotics when there was no infection Compliance of antibiotic selection with current guidelines	Favors intervention
Ng, 2022 [44]	Singapore	Descriptive case series review	12 months	Adults (age 18 years or older), smokers, in the “contemplation” and “preparation” stages of smoking cessation based on the transtheoretical model of smoking behavior	A bedside counseling session by a pharmacist and a follow-up appointment at the outpatient smoking cessation clinic	3	Point-prevalence smoking abstinence rate at 1, 6, and 12 months among the ED SSU smoking cessation service recipients	Potential benefit
Nymoen, 2022 [45]	Norway	Randomized controlled trial	12 months	All patients arriving at the investigated ED, willing to/capable of providing written, informed consent	Systematic medication review by ED clinical pharmacists	2	Proportion of patients with an unplanned contact with the hospital within 12 months after inclusion stay discharge	Neutral
Ogilvie, 2022 [46]	Australia	Randomized controlled trial	NA	Adults (age 18 years or older), referred from the ED for medical admission into the hospital	Pharmacist collaborative prescribing in the ED	1	Not clearly defined	Favors intervention
Stewart, 2021 [47]	USA	Prospective, pilot study	6 months	Adults (age 18 to 60 years old), blood pressure reading > 140/90 mm/Hg, previous diagnosis of hypertension, no consistent primary care relationship (< 1 visit in 6 months), able to provide informed consent	Pharmacist-led TCC provided comprehensive hypertension management under a CPA with ED physicians	3	Change in SBP and DBP from visit to visit throughout the study intervention	Favors intervention
Lineberry, 2021 [48]	USA	Retrospective review	NA	Patients who had a signed discharge prescription on the targeted prescription list	Targeted discharge prescription review service by EMP	2	Intervention rate	Favors intervention
Celikkayalar, 2021 [49]	Finland	Prospective cross-sectional study	NA	Adults admitted to the ED short-term ward	Collaborative Medication Reviews to Identify Inappropriate Prescribing in elderly	4	Not clearly defined	Potential benefit
Castillo, 2021 [50]	USA	Retrospective, observational study	60 days	Patients discharged from the ED with one or more prescriptions during normal hours of EMP coverage, identified by a real-time patient identification tool created in the EHR	EMP review of prescription in EHR and discuss with ED provider if intervention is required, then document interventions	3	Rate, type, and clinical significance of interventions associated with EMP review of ED discharge prescriptions	Potential benefit
Rainess, 2021 [51]	USA	Quasi-experimental-pre-post-interventions study	30 days	Adults discharged home with antibiotics from the ED, and a subsequent positive microbial culture result	The clinical pharmacist reviewed the callback culture and made recommendations to the midlevel provider based	2	Composite outcome of optimal antimicrobial therapy that included optimal antibiotic, optimal dose, and optimal duration of the definitive antibiotic therapy	Favors intervention
Olson, 2020 [52]	USA	Retrospective study	30 days after discharge	Adults discharged from ED and subsequently required an intervention based on the final urine culture or STI assay result	Pharmacist-initiated culture review process	2	Change in time from final culture result to patient contact by an advanced practice provider in definitive urinary tract infection antibiotic therapy	Favors intervention
Roels, 2020 [53]	USA	Descriptive study	365 days	Patients discharged from the ED with a subsequent positive microbial culture result	CPA for pharmacists to follow positive cultures	2	Number of cases reviewed and acted upon independently by the ED pharmacists using the CPA	Potential benefit
Lim, 2020 [54]	Australia	Retrospective case series review	180 days	Patients (adults) with newly diagnosed lower limb DVT, discharged from ED with a “Rivaroxaban after-hours packs”	A “Rivaroxaban after-hours pack” program that provides 24-h, round-the-clock drug supply and guidance to clinicians via a decision-support computer tool	2	Number of patients with prescriptions not complying with recommendations related to bleeding and thrombosis Incidences of bleeding and clot extension Admissions for hospital DVT treatment (study period vs preceding 30 days) Mean hospital contact time for patients	Favors intervention
Tweedle, 2020 [55]	USA	Observational pre-post retrospective study	NA	Patients (age 18 years or younger), discharged home from the ED, with subsequent positive urine and genital culture results	Review of positive cultures by clinical pharmacists	2	Time from positive culture result to time to notification of patient/family	Mixed results
Wu, 2020 [56]	USA	Retrospective observational study	NA	Patients (any age) presented to the ED and had positive microbial culture results, or abnormal, out-of-reference-value laboratory results	Pharmacist-driven emergency department laboratory follow-up and antimicrobial management program	3	Number of interventions made by pharmacists	Potential benefit
Houlind, 2020 [57]	Denmark	Longitudinal feasibility study	30 days after discharge	Older adults (age 65 years or older), admitted to the medical ED	A collaborative medication review, reconciliation, and deprescribing intervention in an ED	2	Change in the Medication Appropriateness Index score from hospital admission to 30 days after discharge	Favors intervention
Layman, 2020 [58]	USA	Retrospective propensity-matched analysis	30 days after discharge	Veterans visiting for HF or COPD exacerbation, or requiring quick follow-up for issues such as insulin titration, blood pressure management, and laboratory monitoring, and veterans seen in the ED who did not have an assigned PCP	Pharmacist-led TCC using clinical pharmacy specialists to perform comprehensive medication management	2	Reduction in the 30-day readmission rates Number of patients readmitted for COPD Number of patients readmitted for HF Average time between discharge and TCC visit Number of medication-related problems identified Pharmacist interventions per patient	Neutral
Pearson, 2020 [59]	USA	Retrospective pre-post intervention	30- and 90-day post-ED discharge	Older adults (age 65 years or older), discharged from ED after a < 24-h stay, and considered high risk for readmission (defined as those with a historical diagnosis of HF/COPD based on registry enrollment, or an additional ED visit in the previous six months)	Post-ED discharge telephonic outreach and assessment by a clinical pharmacist	3	The proportion of patients with at least one repeat ED visit, hospitalization, or death within 30 days of ED discharge	Neutral
Kitchen, 2020 [60]	Canada	Quality improvement initiative	12 months	Adults (19 years or older), presenting to ED during clinical pharmacist working hours, are categorized as at high-risk of presenting with an ADE according to a validated decision rule	Medication review by clinical pharmacists for ED patients with a high risk of presenting with an ADE	4	Differences in the total number of primary care physician visits per 1000 patients per month,12 months following the index visit	Neutral
Shealy, 2020 [61]	USA	Pre-post implementation cohort study	30 days	Adults discharged from ED with positive culture and/or positive STI rapid diagnostic technology result	A live-alert system was implemented for all cultures and STI Rapid Diagnostic Technology updates, which a pharmacy resident reviewed	3	Time from ED discharge to result review Time from ED discharge to completion of outpatient follow-up	Favors intervention
Loborec, 2020 [62]	USA	Pre-post retrospective cohort	90 days	Adults discharged from ED with subsequent positive microbiologic tests (blood/ urine/ skin and soft tissue culture result, viral panels, and STI tests) finalized during the study pharmacist’s work hours	Privileged ED Specialty Practice Pharmacists review and act on microbiologic test results routed within EHR independently of a physician	3	Time to documentation of attempted patient notification Number of Erroneous interventions (including incorrect, missed, and unnecessary interventions)	Mixed results
Eswaran, 2020 [63]	USA	NA	NA	No strict eligibility criteria for patients to receive a THN kit; decision left to the individual provider’s discretion	ED-based THN program	2	Number of ED visits for opioid overdose Number of THN kits dispensed	Potential benefit
Giruzzi, 2020 [64]	USA	Retrospective, quasi-experimental study	30 days post-discharge	Adults discharged from ED with a subsequent positive microbial culture result from any source	Antimicrobial Stewardship Pharmacist Culture Review Service	2	Median time to antimicrobial therapy modification in patients discharged with culture results and antimicrobial therapy that were inadequate	Favors intervention
Cabilan, 2019 [65]	Australia	Quasi-experimental study	48 h	Adults discharged home from the ED short stay unit with newly prescribed medications (not prescribed previously or in the last 12 months)	Pharmacist-led discharge medication counseling	2	Difference in satisfaction with information (measured using SIMS)	Favors intervention
Schwab, 2019 [66]	USA	Prospective cohort study	6 months	Adults presenting to ED during multidisciplinary team working hours, with a diagnosis of non-valvular atrial fibrillation	Multidisciplinary anticoagulation initiative in the ED	2	Change in the proportion of patients on guideline-based DOAC therapy at admission and discharge	Favors intervention
Fay, 2019 [67]	USA	Retrospective quasi-experimental study	72 h post ED and 30 days for hospital admission	Patients (adults) discharged from urgent care units with subsequent positive urine or wound culture results	Pharmacist-led culture follow-up program	3	Total guideline-concordant antibiotic prescribing	Favors intervention
Southerland, 2011 [68]	USA	Prospective observational feasibility study	10 days	Adults (≥ 50 years old), discharged home from ED with at least one new, non-schedule II prescription medication, Mondays through Wednesdays from 11 am to 7 pm	Inter-professional transitions of care services for older adults discharged from ED	3	Current disease state status and medication use at days 3 and 10	Uncertain (no control, and deemed not feasible)
Lacy, 2018 [69]	USA	Non-randomized study	30 days after discharge	Adults admitted to ED who receive primary care at a network clinic staffed with ambulatory pharmacy services	Phone calls by pharmacists within 7 days of ED discharge	2	Primary medication nonadherence following ED visit (patients who had not picked up their medications at the time of the call) Number of medication discrepancies Type of medication discrepancies Interventions resulting from follow-up phone calls	Potential benefit
Perrín, 2018 [70, 77]	Spain	Randomized clinical trial	365 days	Older adults (older than 65 years), presenting to ED, managed at observation areas, taking ≥ 1 medication in the outpatient setting for one chronic condition (defined as conditions lasting > six months)	Pharmacists review the chronic medications of the patients and identify any PIPs according to the STOPP-START criteria	2	The composite of visits to the Emergency Department and all-cause hospital admissions measured as visits at 3, 6, and 12 months (I vs C)	Neutral
Chu, 2017 [71]	USA	Retrospective chart review	90 days	Adults diagnosed with acute VTE and discharged directly from the ED for home management with rivaroxaban during hours of ED pharmacist coverage	ED pharmacists provided extensive counselling to patients on the Rivaroxaban 30-day Starter Pack™, which was filled through the on-site outpatient pharmacy	2	Overall 90-day readmission 90-day readmission rates due to treatment failure or non-adherence Medication adherence beyond the first month from discharge	Neutral
DiRenzo, 2017 [72]	USA	Prospective, observational cohort study	180 days	Adults diagnosed with low-risk VTE in the ED, treated with rivaroxaban, and discharged without hospital admission	Pharmacist-led clot clinic for management of low-risk VTE on rivaroxaban	3	Composite occurrence of anticoagulation treatment-related complications (defined as an episode of major bleeding, recurrent thromboembolic event, or fatal event due to either bleeding or thromboembolism) within 6 months of the original VTE diagnosis	Neutral
Hohl, 2017 [73]	Canada	Pragmatic quasi-randomized design	30 days post-discharge	Adults (age 19 years or older), at high-risk of ADEs, based on the patients’ age, comorbid conditions, recent antibiotic use, and recent medication changes at triage, presenting when a medication review pharmacist was on duty	Clinical Pharmacist-led Systematic Medication Review	2	Number of days spent in-hospital during the 30-day follow-up period	Neutral
Zdyb, 2017 [74]	USA	Retrospective medical record analysis	90 days	Patients (adults) discharged from ED with a new anticoagulant prescription (including LMWHs, warfarin, and DOACs) for any indication	Anticoagulation discharge education provided by ED pharmacists	3	The need for intervention upon callback by the patient	Favors intervention
Hohner, 2016 [75]	USA	Case Study	30 days	Adults presenting ED for an exacerbation of asthma, COPD, or HF, discharged home from ED, established care with a PCP in an adult internal medicine clinic with ambulatory pharmacy services	A TOC pharmacist-led program targeting patients who arrived at the ED with the chief complaint of asthma exacerbation, COPD, or HF	4	Number of successful follow-ups: Attended pharmacist follow-up appointment Mean time from ED visit to follow-up (days between ED visit and follow-up appointment) 30-day ED revisits	Potential benefit
Lingenfelter, 2016 [76]	USA	Retrospective review	No follow-up	Adults discharged home from ED with uncomplicated UTI for outpatient treatment, with subsequent positive urine culture result (> 100 000 CFU/mL of bacterial growth)	ED pharmacist urine culture review	2	Percentage of inappropriately treated urine cultures that were identified by ED pharmacist Percentage of inappropriately treated urine cultures successfully intervened upon by ED pharmacist	Potential benefit
Okere, 2015 [78]	USA	Prospective randomized cohort study	90 days	Any patient (child or adult) presenting to the ED during weekdays, 8:00 am to 4:00 pm. Analysis was restricted to adults (age ≥ 18 years), and the subpopulation taking ≥ 1 prescribed daily medication at the time of the index ED visit	Medication therapy management and reconciliation service from a pharmacist in collaboration with the ED physician	2	Primary care visits Urgent care visits Any ED visits Average number of ED visits (rate/ 1000 persons)	No difference
Briggs, 2015 [79]	Australia	Stratified randomized controlled study	120 days	Older adults (older than 70 years), living at home, taking ≥ five medications	Emergency Department Medication Reconciliation	2	Risk of admission	Favors intervention
Falconieri, 2014 [80]	USA	Retrospective analysis	30 days	Adults admitted and discharged from the ED with acute uncomplicated DVT and anticoagulation	The FAST (Facilitating Anticoagulation for Safer Transitions) DVT discharge program	2	Completion of follow-up appointments Ability of patients to afford/ access their anticoagulant Medication adherence Occurrence of adverse drug events or side effects Hospital admission related to DVT	Potential benefit
Dumkow, 2014 [81]	USA	Quasi-experimental pre-post study	30 days	Adults discharged home with a positive blood or urine culture result	Culture follow-up implemented using computerized decision-support software and a multidisciplinary team of pharmacists and emergency physician staff	1	Composite of ED revisit within 72 h of index ED discharge or hospital admission within 30 days of index ED discharge	Neutral
Angoulvant, 2013 [82]	France	Randomized controlled trial	14 days for the primary outcome	Children (age 1 month to 6 years), discharged from ED with an oral antibiotic prescription for an acute respiratory or urinary tract infection	Face-to-face therapeutic education session for patients on antibiotic use	1	Patient satisfaction at day 14	Favors intervention
Cesarz, 2013 [83]	USA	Prospective observational study	NR	Patients (adult and pediatric) discharged from the ED with a discharge prescription	ED pharmacist review of discharge prescriptions for patients discharged from the emergency department	2	Intervention rates Types of Intervention	Potential benefit
Davis, 2012 [84]	USA	Pilot study	90 days post-enrollment	Newly diagnosed DVT, discharged from ED to outpatient setting on oral and parenteral anticoagulation	Pharmacists-led DVT pilot program in the emergency department	2	Length of ED stay Percentage of patients who attended the follow-up appointments Number of patients who experienced recurrent DVT Number of patients who experienced major bleeding	Potential benefit
Randolph, 2011 [85]	USA	Retrospective evaluation	NR	Patients discharged from ED with a subsequent culture result (any culture)	ED pharmacist involvement in antimicrobial therapy was focused on the review of culture samples drawn in the ED, as well as empiric antibiotic selection	2	Rates of antimicrobial regimen modifications before and after implementation of a pharmacist-managed ED culture review procedure ED readmissions within 96 h of ED discharge Reasons for ED readmissions	Favors intervention
Dip, 2010 [86]	Australia	Prospective evaluation	NR	Older adults (aged ≥ 65 years with a chronic disease or ≥ 70 years without a chronic disease), presenting during the specialist aged care pharmacist working hours, with an Australian Triage Category score of ≥ 2, and fluent in English	Aged Care Pharmacist in the EM to assess elderly patients	2	Efficiency and effectiveness of service Hospital presentation within 14 and 28 days Acceptability of the service Patient and health care professional satisfaction surveys	Mixed results

ADE: Adverse drug events, AMU: Acute medical unit, BPMH: Best-possible medication history, CPA: Collaborative practice agreement, COPD: Chronic obstructive pulmonary disease, DOACs: Direct oral anticoagulants, DVT: Deep vein thrombosis, ED: Emergency Department, EHR: Electronic health record, EMP: Emergency medicine pharmacist, ESBL: Extended spectrum beta-lactamases, GEDI: Geriatric Emergency Department Intervention, HF: Heart failure, LMWH: Low molecular weight heparin, MDR: Multidrug resistant, MRSA: Methicillin-resistant Staphylococcus aureus, PCP: primary care provider, PIP: Potentially inappropriate prescriptions, PPMC: Partnered pharmacist medication charting, SANE: Sexual Assault Nurse Examiner, SIMS: Satisfaction with Information about Medicines Scale, STI: Sexually transmitted infection, TCC, Transitional care clinic, THN, Take home naloxone, TOC: Transition of care, UTI: Urinary tract infection, VRE: Vancomycin-resistant Enterococcus, VTE: Venous thromboembolism

ᵃIntervention intensity was defined as the number of distinct pharmacy-supported transitional care activities included in each intervention package. Activities included: medication reconciliation (when part of a broader package), comprehensive medication management (CMM), discharge planning or care coordination, post-discharge follow-up, medication or self-management education, and medication acquisition assistance. Each activity was counted once; e.g., interventions with both medication reconciliation and CMM were scored as 2, whereas CMM alone was scored as 1

### Intervention development, characteristics, and implementation

Table 3 describes the completeness of reporting across the TIDieR checklist items. All studies (n = 64) reported the name of or a phrase that described the intervention. As illustrated in Table 4, the most cited rationales were the opportunity for pharmacists to expand their role (n = 52), followed by gaps in the existing evidence base (n = 50). In contrast, health equity and access to care were the least cited reasons (n = 8). Moreover, few studies explained how the interventions were developed (34.4%, n = 22). Even when reported, the level of detail varied. For example, some reported establishing collaborative practice agreements to facilitate pharmacists' roles, while others only noted stakeholder involvement in the development process. Notably, only one study linked its intervention to theory [34].

**Table 3:** Showing the completeness of reporting of the TIDieR items in the included studies

	The TIDieR checklist											
First author, Publication year	Item 1. Brief name	Item 2. Why	Item 3. What (Materials)	Item 4. What (Procedures)	Item 5. Who provided	Item 6. How	Item 7. Where	Item 8. When and how much	Item 9. Tailoring	Item 10. Modifications	Item 11. How well (planned)	Item 12: How well (actual)
Kaucher, 2025 [23]	**Y**	**Y**	**Y**	**Y**	**Y**	**Y**	**Y**	**Y**	*NR*	**Y**	*NR*	**Y**
Boot, 2024 [24]	**Y**	**Y**	**Y**	**Y**	**Y**	**Y**	**Y**	**Y**	*NR*	*NR*	**Y**	*NR*
Reilly, 2024 [25]	**Y**	**Y**	**Y**	**Y**	NR	**Y**	**Y**	**Y**	*NR*	*NR*	*NR*	**Y**
Lee, 2024 [26]	**Y**	**Y**	**Y**	**Y**	**Y**	**Y**	**Y**	**Y**	*NR*	*NR*	**Y**	*NR*
Selman, 2024 [27]	**Y**	**Y**	**Y**	**Y**	*NR*	**Y**	**Y**	**Y**	*NR*	*NR*	*NR*	*NR*
Martínez, 2024 [28]	**Y**	**Y**	**Y**	**Y**	*NR*	**Y**	**Y**	**Y**	*NR*	*NR*	**Y**	**Y**
Maleki, 2024 [29]	**Y**	**Y**	**Y**	**Y**	*NR*	*NR*	**Y**	**Y**	*NR*	*NR*	**Y**	*NR*
Sofeso, 2024 [30]	**Y**	**Y**	**Y**	**Y**	*NR*	**Y**	**Y**	**Y**	*NR*	*NR*	*NR*	**Y**
Tran-Nguyen, 2024 [31]	**Y**	**Y**	**Y**	**Y**	*NR*	**Y**	*NR*	**Y**	*NR*	*NR*	*NR*	NR
Atey, 2024 [32]	**Y**	**Y**	**Y**	**Y**	**Y**	**Y**	**Y**	**Y**	*NR*	*NR*	**Y**	**Y**
Wang, 2024 [33]	**Y**	**Y**	**Y**	**Y**	**Y**	**Y**	**Y**	**Y**	*NR*	**Y**	*NR*	*NR*
Kofoed, 2023 [34]	**Y**	**Y**	**Y**	**Y**	*NR*	**Y**	**Y**	**Y**	*NR*	*NR*	**Y**	*NR*
Pham, 2023 [35]	**Y**	**Y**	**Y**	**Y**	*NR*	**Y**	**Y**	**Y**	*NR*	*NR*	*NR*	*NR*
Jovevski, 2023 [36]	**Y**	**Y**	**Y**	**Y**	**Y**	**Y**	**Y**	**Y**	*NR*	*NR*	*NR*	*NR*
Andrade, 2023 [37]	**Y**	**Y**	**Y**	**Y**	**Y**	**Y**	**Y**	**Y**	*NR*	*NR*	**Y**	*NR*
Dunn, 2023 [38]	**Y**	**Y**	**Y**	**Y**	*NR*	**Y**	*NR*	**Y**	*NR*	*NR*	*NR*	**Y**
Atey, 2023 [39, 40]	**Y**	**Y**	**Y**	**Y**	**Y**	**Y**	**Y**	**Y**	*NR*	*NR*	**Y**	*NR*
Atey, 2023 [41]	**Y**	**Y**	**Y**	**Y**	**Y**	**Y**	**Y**	**Y**	*NR*	*NR*	**Y**	*NR*
Zhao, 2023 [42]	**Y**	**Y**	**Y**	**Y**	*NR*	*NR*	**Y**	**Y**	*NR*	*NR*	*NR*	*NR*
Benson, 2023 [43]	**Y**	**Y**	**Y**	**Y**	*NR*	**Y**	**Y**	**Y**	*NR*	*NR*	*NR*	*NR*
Ng, 2022 [44]	**Y**	**Y**	*NR*	**Y**	**Y**	**Y**	**Y**	**Y**	*NR*	*NR*	**Y**	*NR*
Nymoen, 2022 [45]	**Y**	**Y**	**Y**	**Y**	**Y**	**Y**	**Y**	**Y**	*NR*	**Y**	**Y**	*NR*
Ogilvie, 2022 [46]	**Y**	**Y**	**Y**	**Y**	**Y**	**Y**	**Y**	**Y**	*NR*	*NR*	**Y**	*NR*
Stewart, 2021 [47]	**Y**	**Y**	**Y**	**Y**	*NR*	**Y**	**Y**	**Y**	**Y**	*NR*	*NR*	**Y**
Lineberry, 2021 [48]	**Y**	**Y**	**Y**	**Y**	*NR*	**Y**	**Y**	**Y**	*NR*	*NR*	*NR*	**Y**
Celikkayalar, 2021 [49]	**Y**	**Y**	**Y**	**Y**	*NR*	**Y**	**Y**	**Y**	*NR*	*NR*	**Y**	*NR*
Castillo, 2021 [50]	**Y**	**Y**	**Y**	**Y**	*NR*	*NR*	**Y**	**Y**	*NR*	*NR*	*NR*	*NR*
Rainess, 2021 [51]	**Y**	**Y**	**Y**	**Y**	**Y**	**Y**	**Y**	**Y**	*NR*	*NR*	*NR*	*NR*
Olson, 2020 [52]	**Y**	**Y**	**Y**	**Y**	*NR*	**Y**	**Y**	**Y**	*NR*	*NR*	*NR*	*NR*
Roels, 2020 [53]	**Y**	**Y**	**Y**	**Y**	*NR*	*NR*	**Y**	**Y**	*NR*	*NR*	*NR*	**Y**
Lim, 2020 [54]	**Y**	**Y**	**Y**	**Y**	*NR*	**Y**	*NR*	**Y**	*NR*	*NR*	*NR*	**Y**
Tweedle, 2020 [55]	**Y**	**Y**	**Y**	**Y**	**Y**	**Y**	**Y**	**Y**	*NR*	*NR*	*NR*	*NR*
Wu, 2020 [56]	**Y**	**Y**	**Y**	**Y**	**Y**	**Y**	**Y**	**Y**	*NR*	*NR*	**Y**	*NR*
Houlind, 2020 [57]	**Y**	**Y**	**Y**	**Y**	*NR*	**Y**	**Y**	**Y**	*NR*	*NR*	*NR*	**Y**
Layman, 2020 [58]	**Y**	**Y**	**Y**	**Y**	**Y**	**Y**	**Y**	**Y**	**Y**	**Y**	*NR*	*NR*
Pearson, 2020 [69]	**Y**	**Y**	**Y**	**Y**	*NR*	**Y**	**Y**	**Y**	*NR*	*NR*	*NR*	**Y**
Kitchen, 2020 [60]	**Y**	**Y**	**Y**	**Y**	**Y**	**Y**	**Y**	**Y**	*NR*	*NR*	*NR*	*NR*
Shealy, 2020 [61]	**Y**	**Y**	**Y**	**Y**	**Y**	**Y**	**Y**	**Y**	*NR*	*NR*	*NR*	**Y**
Loborec, 2020 [62]	**Y**	**Y**	**Y**	**Y**	**Y**	**Y**	**Y**	**Y**	*NR*	*NR*	**Y**	**Y**
Eswaran, 2020 [63]	**Y**	**Y**	**Y**	**Y**	**Y**	**Y**	**Y**	**Y**	*NR*	*NR*	**Y**	*NR*
Giruzzi, 2020 [64]	**Y**	**Y**	**Y**	**Y**	**Y**	**Y**	**Y**	**Y**	*NR*	*NR*	*NR*	**Y**
Cabilan, 2019 [65]	**Y**	**Y**	**Y**	**Y**	*NR*	**Y**	**Y**	**Y**	*NR*	*NR*	*NR*	*NR*
Schwab, 2019 [66]	**Y**	**Y**	**Y**	**Y**	*NR*	**Y**	**Y**	**Y**	**Y**	**Y**	*NR*	*NR*
Fay, 2019 [67]	**Y**	**Y**	**Y**	**Y**	**Y**	**Y**	**Y**	**Y**	*NR*	*NR*	*NR*	**Y**
Southerland, 2011 [68]	**Y**	**Y**	**Y**	**Y**	*NR*	**Y**	**Y**	**Y**	*NR*	**Y**	*NR*	**Y**
Lacy, 2018 [69]	**Y**	**Y**	**Y**	**Y**	**Y**	**Y**	*NR*	**Y**	*NR*	*NR*	**Y**	**Y**
Perrín, 2018 [70, 77]	**Y**	**Y**	**Y**	**Y**	*NR*	**Y**	**Y**	**Y**	*NR*	*NR*	*NR*	**Y**
Chu, 2017 [72]	**Y**	**Y**	**Y**	**Y**	*NR*	**Y**	**Y**	**Y**	*NR*	*NR*	*NR*	*NR*
DiRenzo, 2017 [72]	**Y**	**Y**	**Y**	**Y**	*NR*	**Y**	**Y**	**Y**	*NR*	**Y**	**Y**	*NR*
Hohl, 2017 [73]	**Y**	**Y**	**Y**	**Y**	**Y**	**Y**	**Y**	**Y**	*NR*	*NR*	**Y**	*NR*
Zdyb, 2017 [74]	**Y**	**Y**	**Y**	**Y**	*NR*	**Y**	**Y**	**Y**	*NR*	*NR*	**Y**	**Y**
Hohner, 2016 [75]	**Y**	**Y**	**Y**	**Y**	**Y**	**Y**	**Y**	**Y**	*NR*	*NR*	**Y**	**Y**
Lingenfelter, 2016 [76]	**Y**	**Y**	**Y**	**Y**	*NR*	**Y**	**Y**	**Y**	*NR*	*NR*	*NR*	**Y**
Okere, 2015 [78]	**Y**	**Y**	**Y**	**Y**	*NR*	**Y**	**Y**	**Y**	*NR*	*NR*	**Y**	*NR*
Briggs, 2015 [79]	**Y**	**Y**	**Y**	**Y**	*NR*	**Y**	**Y**	**Y**	*NR*	*NR*	*NR*	Y
Falconieri, 2014 [80]	**Y**	**Y**	**Y**	**Y**	**Y**	**Y**	*NR*	**Y**	*NR*	**Y**	**Y**	**Y**
Dumkow, 2014 [81]	**Y**	**Y**	**Y**	**Y**	**Y**	**Y**	**Y**	**Y**	*NR*	*NR*	*NR*	*NR*
Angoulvant, 2013 [82]	**Y**	**Y**	**Y**	**Y**	**Y**	**Y**	**Y**	**Y**	*NR*	*NR*	**Y**	**Y**
Cesarz, 2013 [83]	**Y**	**Y**	**Y**	**Y**	**Y**	**Y**	**Y**	**Y**	*NR*	*NR*	**Y**	**Y**
Davis, 2012 [84]	**Y**	**Y**	**Y**	**Y**	**Y**	**Y**	**Y**	**Y**	**Y**	**Y**	**Y**	**Y**
Randolph, 2011 [85]	**Y**	**Y**	**Y**	**Y**	*NR*	**Y**	*NR*	**Y**	*NR*	*NR*	*NR*	*NR*
Dip, 2010 [86]	**Y**	**Y**	**Y**	**Y**	**Y**	**Y**	**Y**	**Y**	*NR*	*NR*	*NR*	*NR*

Y: Yes; NR: Not Reported

**Reference**: Hoffmann TC, Glasziou PP, Boutron I, Milne R, Perera R, Moher D, et al. Better reporting of interventions: template for intervention description and replication (TIDieR) checklist and guide. BMJ. 2014;348:g1687

**Table 4:** Summary of the reported rationales for the pharmacy-supported transition of care interventions in the emergency department

Rationale	n (%)	Example
Opportunity for pharmacists to expand their role	52 (81.25%)	“Despite these initiatives, pharmacists in Australia have a limited role in prescribing medications for patients being discharged from [the] hospital.” [21]
Gaps in the existing evidence base	50 (78.12%)	“Prior studies have focused on outpatient interventions or inpatient medication reconciliation and have yielded mixed results in fall reduction. There is sparse data on ED-based deprescribing programs, thus necessitating the need for additional research.”[22]
ED-specific challenges and opportunities	49 (76.56%)	“The emergency department (ED) is at an increased risk for medication errors given the high patient volumes, lack of familiarity with the patient compared to their primary care provider, and rapid turnover rates.”[25]
Supporting evidence showing benefit of similar interventions	45 (70.31%)	“Previous studies have shown that pharmacist involvement leads to a decrease in time to review a positive culture, time to patient notification, ED revisits, as well as improvement in appropriateness of changes in therapy” [19]
Policy, guideline, and institutional drivers	25 (39.06%)	“In 2017, the Joint Commission established the requirement for hospitals and nursing homes to have an antimicrobial stewardship program (ASP)” [56]
Health system efficiency and cost considerations	21 (32.81%)	“Unplanned hospital readmission is a safety and quality healthcare measure, conferring significant costs to the healthcare system.” [26]
Equity and access to care	8 (12.50%)	“ED physicians in urban, under-resourced medical settings encounter a significant number of patients in the community who are utilizing the ED for primary care needs, such as chronic HTN management” [42]

A wide range of physical and informational materials were used across interventions, with electronic health records being most consistently utilized. Many studies incorporated telephone, letters, or fax for follow-up. Several relied on clinical guidelines (e.g., STOPP/START criteria, IDSA guidelines), medication lists, and culture reports to guide therapy. A few provided unique materials such as starter medication packs, demonstration kits, and access to national prescribing databases [23, 54, 63, 71, 84]. Detailed procedures are provided in Table S6. Intervention intensity ranged from 1 to 4, with a median of 2 (Table 2). Most services focused on CMM and discharge planning, including timely communication and care coordination among team members, each reported in 60.9% of studies (n = 39). Medication reconciliation was conducted in 40.6% (n = 26), while post-discharge follow-up involving monitoring and managing symptoms was reported in 39% (n = 25). Medication and self-management education were described in 32.8% of studies. Only two studies (3.1%) included medication acquisition assistance. Regarding transitions of care touchpoints, interventions were most implemented post-discharge (n = 35, 54.7%), followed by arrival to the ED (n = 27, 42.2%), discharge from the ED (n = 15, 24.4%), intra-hospital transfer (n = 5, 7.8%), and inter-hospital transfer (n = 1, 1.56%).

### Intervention delivery and location

The interventions were mostly delivered by pharmacists (n = 52, 81.3%), followed by teams of pharmacy residents and/or students supervised by pharmacists (n = 9, 14.1%), and pharmacy residents alone (n = 3, 4.7%). Reporting on pharmacists’ characteristics was inconsistent, with considerable variability in the level of detail provided, especially for years of experience and specialization areas. (Table S6). Nearly half (n = 30) did not adequately describe the pharmacists’ backgrounds, training, or credentials, often using vague terms like “experienced pharmacists”. Among those, 25% reported post-graduate residency training, 8% reported board certification, and 6% reported extended privileging. Few studies detailed intervention-specific training or required formal competency assessments. One study [39] provided detailed descriptions of the credentialing pathway for partnered pharmacist medication charting and the training received via resources like the SHPA ClinCAT tool and OSCE credentialing assessments. Most studies (n = 63) provided clear descriptions of delivery processes and explicitly described procedural data on how the intervention were delivered (Table S6). Pharmacists delivered the intervention independently in 23 studies (36%). In the remaining 41, delivery was collaborative with other healthcare professionals, most commonly physicians, who viewed or approved of pharmacists’ recommendations. Interventions were mostly delivered at a single center (n = 53). Moreover, most interventions were implemented in acute care ED units (n = 58), with only 6 studies in ED medical wards, short stay, or observation units. Concerning the level of care, 56.2% of the interventions were in tertiary hospital-based EDs, followed by community hospital-based EDs (15.6%), and secondary hospital-based EDs (3.1%). These EDs varied in patient volumes, with annual attendances ranging from 11,900 to 185,000 patients. Only 25% of the studies reported intervention durations, which ranged from 5 to 75 min per intervention (Table S7).

### Intervention tailoring, modifications, and fidelity

Tailoring and modifications to the original intervention were infrequently reported across the studies (Table S7). Interventions were individualized to address patient-specific needs and barriers (n = 4). For instance, financial waivers were considered on a case-by-case basis for patients unable to afford essential medications, involving collaboration among pharmacists, physicians, and hospital administrators [66, 84]. Intervention modifications included expanding services (n = 2), adjusting eligibility criteria, and revising protocols to address staffing limitations or low recruitment. Some were modified to improve access (n = 3). Fewer than half of the studies described planned measures to ensure fidelity in delivering their interventions (n = 27), with 10.9% reporting multiple measures. Of those, standardization of implementation protocol or guides was the most common measure (n = 15). An independent review of recommendations was also reported. Similarly, 43.7% of the studies included information on the actual fidelity of the interventions. Among those, the reported indicators were loss to follow-up rate (n = 12), along with the percentages of completed interventions (n = 10), pharmacists’ recommendations that were accepted by prescribers (n = 6), and recommendations determined as appropriate by reviewers (n = 2). Notably, loss to follow-up rates ranged from 2.5% [48] to 76% [23]. Likewise, prescribers’ acceptance of pharmacists’ recommendations varied, ranging between 27 [70] and 82% [28].

### Outcomes (interventional studies)

**Fig. 2:**
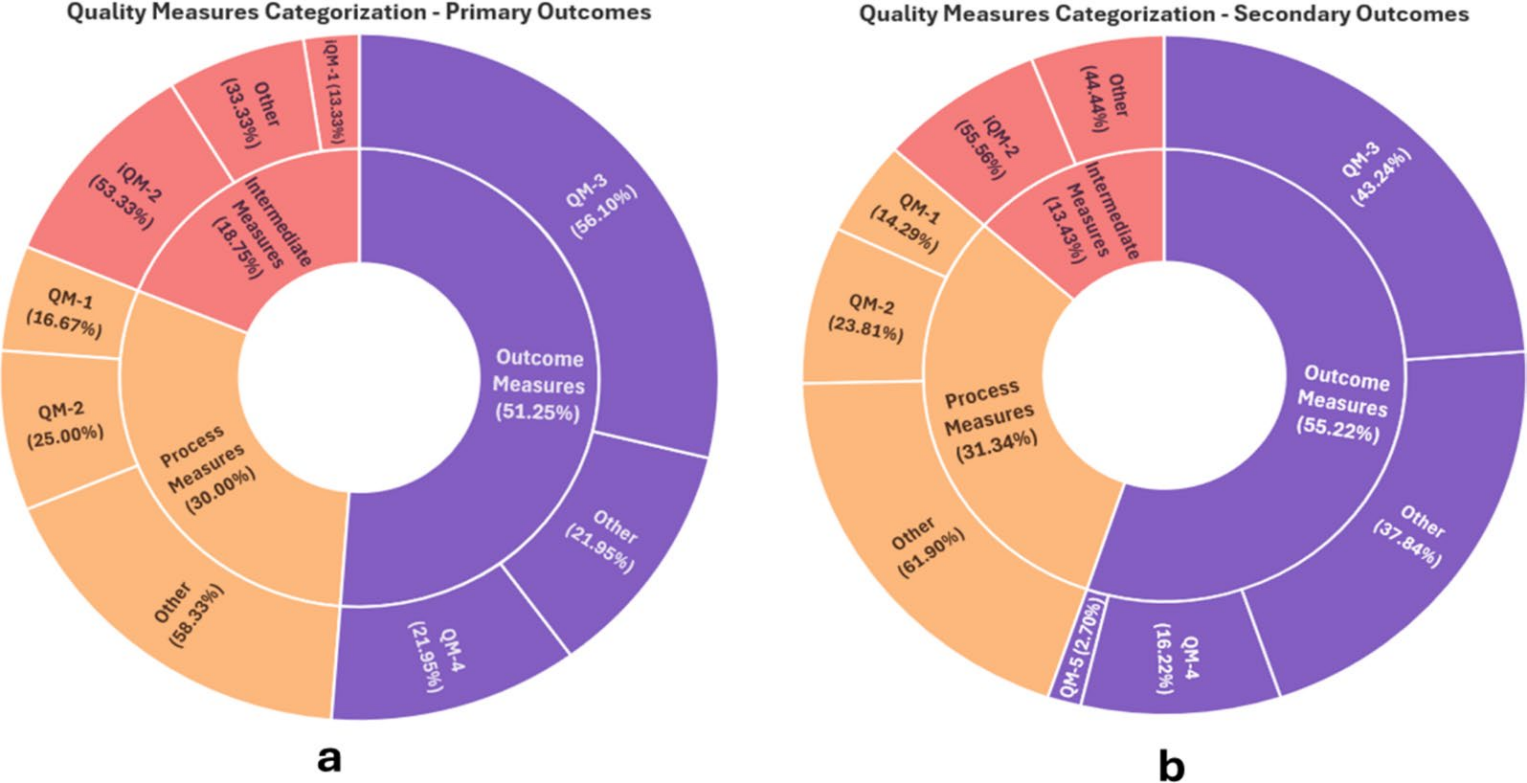
The proportions of reported measures across the included studies for primary (**a**) and secondary (**b**) outcomes

Figure 2 illustrates the proportions of reported quality measures across the sample using the ACCP framework. For both primary (n = 41, 51.2%) and secondary outcomes (n = 37, 55.2%), most measures assessed final outcomes rather than process (n = 24, 30.0%; n = 21, 31.34%) and intermediate outcomes (n = 15, 18.75%; n = 9, 13.43%). Among all outcomes, healthcare utilization measures, particularly readmission rates and primary care follow-up at 30 days, were the most common (n = 23, 28.8%; n = 16, 23.9%).

Pharmacist-led transitional care interventions demonstrated improvement in guideline-concordant antibiotic prescribing (n = 3), antibiotic-free days (n = 1), time from ED discharge to culture result review (n = 1), and time to antibiotics modification (n = 1). Improved medication appropriateness was also reported through diverse measures, including optimization of discharge prescriptions (n = 1), medication error rate (n = 2), potentially inappropriate medication (PIM) deprescribing (n = 2), and adherence to guideline-based oral anticoagulant (OAC) therapy (n = 1). Other positive outcomes included: significant reduction in healthcare utilization (n = 3), patient satisfaction (n = 2), and improvement in blood pressure control (n = 1). Secondary outcomes are reported in Table S8.

## Discussion

In this scoping review, the characteristics and outcomes of pharmacy-supported TOC interventions in EDs, beyond medication reconciliation, were explored. Our findings highlight that while pharmacists’ role in ED-based transitional care is expanding, the existing evidence is heterogeneous, insufficiently reported in important domains, and limited in its generalizability and ability to demonstrate long-term impact.

### Summary of key findings

A wide variety of interventions were identified, ranging from medication reconciliation and medication optimization to post-discharge follow-up by phone or letter. Most interventions focused on CMM and discharge planning, aligning with the proposed ideal practice model for transitional care services [87, 88]. Of note, no studies reported interventions targeting transfer to another healthcare facility [64], and only five focused on intra-hospital transfer. As per the WHO report on medication safety during transitions, 62% of patients experience medication discrepancies during intra-hospital transition [88].

Moreover, our review shows that most interventions incorporated multiple components, with 90.6% (n = 58). involving two or more activities. This finding echoes the systematic review by Alhmoud et al. [89]. Interestingly, a network meta-analysis focused on hospital-to-community interventions found that interventions of low to medium intensity (1–7 components) were more effective in reducing healthcare utilization than those of higher intensity (8 + components) [90]. However, their analysis included a wide range of components delivered by different providers, raising uncertainty about whether findings can be attributed solely to intervention intensity.

Furthermore, interventions often targeted high-risk patients (e.g., older adults or patients with polypharmacy/comorbidities). This is reasonable considering that such patients are at a higher risk of medication-related readmissions [91]. Still, despite being vulnerable to errors during ED visits [92], pediatric populations were underrepresented in our review (n = 3) [55, 82, 83], highlighting an opportunity for developing and evaluating initiatives targeting this group.

Regarding provider roles, most interventions (64%, n = 41) described pharmacists collaborating with other healthcare providers, emphasizing the importance of multidisciplinary care in transitions [22]. Nonetheless, almost half (n = 30) did not sufficiently report the characteristics and qualifications of intervention providers, limiting insight into the expertise required and potentially compromising replicability. A few studies (n = 11) involved pharmacy residents and students under supervision, highlighting opportunities to engage them in intervention delivery.

In terms of outcomes, healthcare utilization metrics were most frequently reported. This is consistent with previous studies and aligns with the metrics’ growing role as quality indicators for care transitions [22, 89]. However, it is essential to consider the potential of missed health events, as many studies relied on data limited to their own health systems [24, 27, 28, 31, 41, 69, 81]. Evidence suggests that approximately 20–30% of hospital readmissions occur outside the original hospital [93, 94].

### Knowledge gaps

Several key gaps were identified. First, poor reporting of intervention fidelity, tailoring, modification, and development processes limits the replicability of interventions and the interpretation of findings. Also, actual fidelity was rarely and inconsistently reported, making it difficult to determine how consistently interventions were delivered and whether outcomes reflected the intervention itself or variability in implementation. As implementation is central to health service research, more context on how interventions were implemented, including facilitators and barriers, is essential to inform replicability and scalability. Another area of concern is the wide variability in prescribers' acceptance of pharmacists’ recommendations (2.5–76%). For example, Santolaya-Perrin reported acceptance rates from 27 to 53% depending on the site, with significant reductions in ED visits and hospital admissions observed in centers with high acceptance rates [70, 77]. Barriers to prescribers’ uptake of pharmacists’ recommendations in secondary care can include communication methods, priorities mismatch, pharmacists’ presence during decision making, and hierarchies [95]. Those challenges highlight the importance of seeking institutional support when developing ToC interventions and engaging providers and other stakeholders in the process, to foster trust and strengthen multidisciplinary collaboration.

Moreover, most studies' follow-up durations were restricted to short-term outcomes, typically 30 days post-discharge, which may not reflect long-term effects and overestimate benefits [96]. Lastly, equity and accessibility of care were rarely addressed. Most interventions were implemented in tertiary, acute care hospitals in high-income countries (the United States, Australia, and Europe), raising concerns about generalizability to other settings. Understanding how EDs function across countries is important when assessing or implementing ToC services. In countries like the US, Italy, and the Middle East, EDs serve as entry points for both urgent and non-urgent needs [97–100], whereas in systems like the United Kingdom, primary care streaming and triage limit direct ED access for non-urgent cases [101].

### Strengths and limitations of the review

This is the first scoping review exploring pharmacist-supported ToC interventions in ED settings. Our search was comprehensive with a data collection tool adapted from the TIDieR framework, which enabled thorough extraction of intervention characteristics. This review has a few limitations. First, studies that focused exclusively on medication reconciliation as the sole intervention were excluded, as this area is already well established [9, 10], and aim was to capture pharmacists’ roles in transitional care interventions in EDs beyond routine responsibilities. Second, when assessing the TIDieR items, we did not develop criteria for partial compliance, which may have limited the granularity of our evaluation. Lastly, as this is a scoping review, we did not assess the quality of evidence. However, the breadth of included studies and the variability in intervention design and reporting suggest potential for future systematic reviews to evaluate both clinical effectiveness and methodological rigor.

## Conclusion

This scoping review highlights the breadth and complexity of pharmacist-supported transitional care interventions within ED settings. While the growing involvement of pharmacists in ToC services within EDs and short-stay units is evident, critical gaps persist in the reporting and implementation of these interventions. Enhancing the transparency and consistency of reporting interventions is essential to understanding and advancing pharmacist-led ToC services. Furthermore, greater attention should be directed towards underrepresented populations and intra-hospital transitions. All in all, there is a need for future research to systematically examine such initiatives and evaluate their effectiveness and long-term impact.

## Supplementary Information

Below is the link to the electronic supplementary material.

Supplementary file1 (DOCX 138 kb)

## Data Availability

Full data are provided in the supplementary material. Any additional information or clarification is available upon request.
