# Supplementary material for: Characteristics and outcomes of pharmacy-supported transitions of care interventions in emergency departments: a scoping review
Source: Int J Clin Pharm. 2025 Dec 4;48(2):393–423. doi: 10.1007/s11096-025-02057-0 (PMC12992488; doi:10.1007/s11096-025-02057-0)
Supplement: Supplementary file 1 — Supplementary file1 (DOCX 138 kb) [file 11096_2025_2057_MOESM1_ESM.docx]

**Supplementary Materials**

**Title:** Characteristics and Outcomes of Pharmacy-Supported Transitions of Care in Emergency Departments: A Scoping Review

**Journal:** International Journal of Clinical Pharmacy (IJCP)

**Authors:** Eman Alhmoud^1,2^, Waad Elamin^3^, Raja Barazi^,2^, Zeana Alkudsi^2^, Farah Zahrah^2^, Muhammad Abdul Hadi^3*^

1. QU Health Sector, Qatar University, Doha, Qatar
2. Pharmacy Department, Hamad Medical Corporation, Doha, Qatar
3. College of Pharmacy, QU Health Sector, Qatar University, Doha, Qatar

***Corresponding author**

**Muhammad Abdul Hadi**

BPharm MClinPharm PhD PGCertHE FHEA FRSPH

Associate Professor of Clinical Pharmacy and Practice

Graduate Coordinator: PhD in Health Sciences (Clinical and Population Health Research)

Department of Clinical Pharmacy and Practice

College of Pharmacy, QU Health,

Qatar University

Doha, P.O. Box 2713

Qatar

Tel: 00974 4403 5582

Email: [mabdulhadi@qu.edu.qa](mailto:mabdulhadi@qu.edu.qa)

**Table of Contents**

| **Table/Figure** | **Page #** |
| --- | --- |
| **Table S1:** Search strategy for PubMed | 2 |
| **Table S2:** Search strategy for CINAHL | 8 |
| **Table S3:** Search strategy for Embase | 11 |
| **Table S4:** Data Extraction Tool – Part A (Overall Characteristics) | 13 |
| **Table S5:** Data Extraction Tool – Part B ( Reporting of TiDieR Items & Intervention Design), adapted from Hoffman et al. [1] | 14 |
| **Table S6:** Showing the summary of the TIDieR items (1-6) for the pharmacy-supported transitional care interventions in the emergency department | 14 |
| **Table S7:** Showing the summary of the TIDieR items (7-12) for the pharmacy-supported transitional care interventions in the emergency department | 23 |
| **Table S8:** Showing the secondary outcome descriptions of the included studies | 36 |
| **References** | **39** |

***Table S1:*** *Search strategy for PubMed*

| **No.** | **Query** | **Search Details** | **Results** |
| --- | --- | --- | --- |
| #49 | (((((((((Pharmacists[MeSH Terms]) OR (Pharmacy Technicians[MeSH Terms])) OR (Students, Pharmacy[MeSH Terms])) OR (Pharmacy Residencies[MeSH Terms])) OR (Pharmacy[MeSH Terms])) OR (Pharmaceutical Services[MeSH Terms])) OR (Pharmacist*[Title/Abstract])) OR (Pharmacy[Title/Abstract])) AND ((((((((((((((((((((((((((((((((((Continuity of Patient Care[MeSH Terms]) OR (Medication Reconciliation[MeSH Terms])) OR (Medication Review[MeSH Terms])) OR (Medication Therapy Management[MeSH Terms])) OR (Patient Education as Topic[MeSH Terms])) OR (Counseling[MeSH Terms])) OR (Transitions of care[Title/Abstract])) OR (transition of care[Title/Abstract])) OR (transition-of-care[Title/Abstract])) OR (transitions-of-care[Title/Abstract])) OR (TOC[Title/Abstract])) OR (transitional care[Title/Abstract])) OR (Care Transition*[Title/Abstract])) OR (hospital to home[Title/Abstract])) OR (Discharge[Title/Abstract])) OR (Transfer[Title/Abstract])) OR (Admission[Title/Abstract])) OR (Coordination[Title/Abstract])) OR (Continuity of Care[Title/Abstract])) OR (Care Continuity[Title/Abstract])) OR (Handoff[Title/Abstract])) OR (Hand off*[Title/Abstract])) OR (Hand over[Title/Abstract])) OR (Handover[Title/Abstract])) OR (Hands off[Title/Abstract])) OR (Education[Title/Abstract])) OR (Counseling[Title/Abstract])) OR (medication chart[Title/Abstract])) OR (Review[Title/Abstract])) OR (Reconcil*[Title/Abstract])) OR (medication history[Title/Abstract])) OR (therapy management[Title/Abstract])) OR (follow-up[Title/Abstract])) OR (follow up[Title/Abstract]))) AND ((Emergency service, hospital[MeSH Terms]) OR (Emergency[Title/Abstract])) | ("pharmacists"[MeSH Terms] OR "pharmacy technicians"[MeSH Terms] OR "students, pharmacy"[MeSH Terms] OR "pharmacy residencies"[MeSH Terms] OR ("Pharmacy"[MeSH Terms] OR "pharmacies"[MeSH Terms]) OR "pharmaceutical services"[MeSH Terms] OR "pharmacist*"[Title/Abstract] OR "Pharmacy"[Title/Abstract]) AND ("continuity of patient care"[MeSH Terms] OR "medication reconciliation"[MeSH Terms] OR "medication review"[MeSH Terms] OR "medication therapy management"[MeSH Terms] OR "patient education as topic"[MeSH Terms] OR "Counseling"[MeSH Terms] OR "transitions-of-care"[Title/Abstract] OR "transition-of-care"[Title/Abstract] OR "transition-of-care"[Title/Abstract] OR "transitions-of-care"[Title/Abstract] OR "TOC"[Title/Abstract] OR "transitional care"[Title/Abstract] OR "care transition*"[Title/Abstract] OR "hospital to home"[Title/Abstract] OR "Discharge"[Title/Abstract] OR "Transfer"[Title/Abstract] OR "Admission"[Title/Abstract] OR "Coordination"[Title/Abstract] OR "continuity of care"[Title/Abstract] OR "care continuity"[Title/Abstract] OR "Handoff"[Title/Abstract] OR "hand off*"[Title/Abstract] OR "hand over"[Title/Abstract] OR "Handover"[Title/Abstract] OR "hands off"[Title/Abstract] OR "Education"[Title/Abstract] OR "Counseling"[Title/Abstract] OR "medication chart"[Title/Abstract] OR "Review"[Title/Abstract] OR "reconcil*"[Title/Abstract] OR "medication history"[Title/Abstract] OR "therapy management"[Title/Abstract] OR "follow-up"[Title/Abstract] OR "follow-up"[Title/Abstract]) AND ("emergency service, hospital"[MeSH Terms] OR "Emergency"[Title/Abstract]) | 2,948 |
| #48 | (Emergency service, hospital[MeSH Terms]) OR (Emergency[Title/Abstract]) | "emergency service, hospital"[MeSH Terms] OR "Emergency"[Title/Abstract] | 405,449 |
| #47 | Emergency[Title/Abstract] | "Emergency"[Title/Abstract] | 376,697 |
| #46 | Emergency service, hospital[MeSH Terms] | "emergency service, hospital"[MeSH Terms] | 105,715 |
| #45 | space2[Title/Abstract] | "space2"[Title/Abstract] | 32 |
| #44 | (((((((((((((((((((((((((((((((((Continuity of Patient Care[MeSH Terms]) OR (Medication Reconciliation[MeSH Terms])) OR (Medication Review[MeSH Terms])) OR (Medication Therapy Management[MeSH Terms])) OR (Patient Education as Topic[MeSH Terms])) OR (Counseling[MeSH Terms])) OR (Transitions of care[Title/Abstract])) OR (transition of care[Title/Abstract])) OR (transition-of-care[Title/Abstract])) OR (transitions-of-care[Title/Abstract])) OR (TOC[Title/Abstract])) OR (transitional care[Title/Abstract])) OR (Care Transition*[Title/Abstract])) OR (hospital to home[Title/Abstract])) OR (Discharge[Title/Abstract])) OR (Transfer[Title/Abstract])) OR (Admission[Title/Abstract])) OR (Coordination[Title/Abstract])) OR (Continuity of Care[Title/Abstract])) OR (Care Continuity[Title/Abstract])) OR (Handoff[Title/Abstract])) OR (Hand off*[Title/Abstract])) OR (Hand over[Title/Abstract])) OR (Handover[Title/Abstract])) OR (Hands off[Title/Abstract])) OR (Education[Title/Abstract])) OR (Counseling[Title/Abstract])) OR (medication chart[Title/Abstract])) OR (Review[Title/Abstract])) OR (Reconcil*[Title/Abstract])) OR (medication history[Title/Abstract])) OR (therapy management[Title/Abstract])) OR (follow-up[Title/Abstract])) OR (follow up[Title/Abstract]) | "continuity of patient care"[MeSH Terms] OR "medication reconciliation"[MeSH Terms] OR "medication review"[MeSH Terms] OR "medication therapy management"[MeSH Terms] OR "patient education as topic"[MeSH Terms] OR "Counseling"[MeSH Terms] OR "transitions-of-care"[Title/Abstract] OR "transition-of-care"[Title/Abstract] OR "transition-of-care"[Title/Abstract] OR "transitions-of-care"[Title/Abstract] OR "TOC"[Title/Abstract] OR "transitional care"[Title/Abstract] OR "care transition*"[Title/Abstract] OR "hospital to home"[Title/Abstract] OR "Discharge"[Title/Abstract] OR "Transfer"[Title/Abstract] OR "Admission"[Title/Abstract] OR "Coordination"[Title/Abstract] OR "continuity of care"[Title/Abstract] OR "care continuity"[Title/Abstract] OR "Handoff"[Title/Abstract] OR "hand off*"[Title/Abstract] OR "hand over"[Title/Abstract] OR "Handover"[Title/Abstract] OR "hands off"[Title/Abstract] OR "Education"[Title/Abstract] OR "Counseling"[Title/Abstract] OR "medication chart"[Title/Abstract] OR "Review"[Title/Abstract] OR "reconcil*"[Title/Abstract] OR "medication history"[Title/Abstract] OR "therapy management"[Title/Abstract] OR "follow-up"[Title/Abstract] OR "follow-up"[Title/Abstract] | 5,554,044 |
| #43 | follow up[Title/Abstract] | "follow up"[Title/Abstract] | 1,312,364 |
| #42 | follow-up[Title/Abstract] | "follow-up"[Title/Abstract] | 1,312,364 |
| #41 | therapy management[Title/Abstract] | "therapy management"[Title/Abstract] | 2,920 |
| #40 | medication history[Title/Abstract] | "medication history"[Title/Abstract] | 2,144 |
| #39 | Reconcil*[Title/Abstract] | "reconcil*"[Title/Abstract] | 23,522 |
| #38 | Review[Title/Abstract] | "Review"[Title/Abstract] | 2,415,975 |
| #37 | medication chart[Title/Abstract] | "medication chart"[Title/Abstract] | 120 |
| #36 | Counseling[Title/Abstract] | "Counseling"[Title/Abstract] | 94,001 |
| #35 | Education[Title/Abstract] | "Education"[Title/Abstract] | 683,983 |
| #34 | Hands off[Title/Abstract] | "hands off"[Title/Abstract] | 612 |
| #33 | Handover[Title/Abstract] | "Handover"[Title/Abstract] | 2,260 |
| #32 | Hand over[Title/Abstract] | "hand over"[Title/Abstract] | 793 |
| #31 | Hand off*[Title/Abstract] | "hand off*"[Title/Abstract] | 514 |
| #30 | Handoff[Title/Abstract] | "Handoff"[Title/Abstract] | 1,679 |
| #29 | Care Continuity[Title/Abstract] | "care continuity"[Title/Abstract] | 856 |
| #28 | Continuity of Care[Title/Abstract] | "continuity of care"[Title/Abstract] | 10,131 |
| #27 | Coordination[Title/Abstract] | "Coordination"[Title/Abstract] | 154,747 |
| #26 | Admission[Title/Abstract] | "Admission"[Title/Abstract] | 262,824 |
| #25 | Transfer[Title/Abstract] | "Transfer"[Title/Abstract] | 578,445 |
| #24 | Discharge[Title/Abstract] | "Discharge"[Title/Abstract] | 265,260 |
| #23 | hospital to home[Title/Abstract] | "hospital to home"[Title/Abstract] | 1,450 |
| #22 | Care Transition*[Title/Abstract] | "care transition*"[Title/Abstract] | 3,079 |
| #21 | transitional care[Title/Abstract] | "transitional care"[Title/Abstract] | 2,616 |
| #20 | TOC[Title/Abstract] | "TOC"[Title/Abstract] | 10,069 |
| #19 | transitions-of-care[Title/Abstract] | "transitions-of-care"[Title/Abstract] | 1,679 |
| #18 | transition-of-care[Title/Abstract] | "transition-of-care"[Title/Abstract] | 1,279 |
| #17 | transition of care[Title/Abstract] | "transition of care"[Title/Abstract] | 1,279 |
| #16 | Transitions of care[Title/Abstract] | "transitions of care"[Title/Abstract] | 1,679 |
| #15 | Counseling[MeSH Terms] | "counseling"[MeSH Terms] | 50,453 |
| #14 | Patient Education as Topic[MeSH Terms] | "patient education as topic"[MeSH Terms] | 90,072 |
| #13 | Medication Therapy Management[MeSH Terms] | "medication therapy management"[MeSH Terms] | 2,892 |
| #12 | Medication Review[MeSH Terms] | "medication review"[MeSH Terms] | 156 |
| #11 | Medication Reconciliation[MeSH Terms] | "medication reconciliation"[MeSH Terms] | 1,659 |
| #10 | Continuity of Patient Care[MeSH Terms] | "continuity of patient care"[MeSH Terms] | 306,494 |
| #9 | (((((((Pharmacists[MeSH Terms]) OR (Pharmacy Technicians[MeSH Terms])) OR (Students, Pharmacy[MeSH Terms])) OR (Pharmacy Residencies[MeSH Terms])) OR (Pharmacy[MeSH Terms])) OR (Pharmaceutical Services[MeSH Terms])) OR (Pharmacist*[Title/Abstract])) OR (Pharmacy[Title/Abstract]) | "pharmacists"[MeSH Terms] OR "pharmacy technicians"[MeSH Terms] OR "students, pharmacy"[MeSH Terms] OR "pharmacy residencies"[MeSH Terms] OR "Pharmacy"[MeSH Terms] OR "pharmacies"[MeSH Terms] OR "pharmaceutical services"[MeSH Terms] OR "pharmacist*"[Title/Abstract] OR "Pharmacy"[Title/Abstract] | 154,503 |
| #8 | Pharmacy[Title/Abstract] | "Pharmacy"[Title/Abstract] | 60,092 |
| #7 | Pharmacist*[Title/Abstract] | "pharmacist*"[Title/Abstract] | 47,460 |
| #6 | Pharmaceutical Services[MeSH Terms] | "pharmaceutical services"[MeSH Terms] | 84,494 |
| #5 | Pharmacy[MeSH Terms] | "pharmacy"[MeSH Terms] OR "pharmacies"[MeSH Terms] | 17,871 |
| #4 | Pharmacy Residencies[MeSH Terms] | "pharmacy residencies"[MeSH Terms] | 441 |
| #3 | Students, Pharmacy[MeSH Terms] | "students, pharmacy"[MeSH Terms] | 4,893 |
| #2 | Pharmacy Technicians[MeSH Terms] | "pharmacy technicians"[MeSH Terms] | 937 |
| #1 | Pharmacists[MeSH Terms] | "pharmacists"[MeSH Terms] | 23,360 |

***Table S2:*** *Search strategy for CINAHL*

| **Concepts** | **Sub-terms** | **Search options** |
| --- | --- | --- |
| **Pharmacy Personnel** | (MH "Pharmacists+" OR MH "Pharmacy Technicians+" OR MH "Pharmacy Students+" OR MH "Pharmacy Residencies+" OR MH "Pharmacy+" OR MH "Pharmaceutical Services+" OR TI Pharmacist* OR AB Pharmacist* OR TI Pharmacy OR AB Pharmacy) | MH/TI/AB |
| **Transition of care / TOC interventions** | (MH "Continuity of Patient Care+" OR MH "Medication Reconciliation+" OR MH "Medication Review+" OR MH "Medication Therapy Management+" OR MH "Patient Education as Topic+" OR MH "Counseling+" OR TI Transitions of care OR AB Transitions of care OR TI transition of care OR AB transition of care OR TI transition-of-care OR AB transition-of-care OR TI transitions-of-care OR AB transitions-of-care OR TI TOC OR AB TOC OR TI transitional care OR AB transitional care OR TI Care Transition* OR AB Care Transition* OR TI hospital to home OR AB hospital to home OR TI Discharge OR AB Discharge OR TI Transfer OR AB Transfer OR TI Admission OR AB Admission OR TI Coordination OR AB Coordination OR TI Continuity of Care OR AB Continuity of Care OR TI Care Continuity OR AB Care Continuity OR TI Handoff OR AB Handoff OR TI Hand off* OR AB Hand off* OR TI Hand over OR AB Hand over OR TI Handover OR AB Handover OR TI Hands off OR AB Hands off OR TI Education OR AB Education OR TI Counseling OR AB Counseling OR TI medication chart OR AB medication chart OR TI Review OR AB Review OR TI Reconcil* OR AB Reconcil* OR TI medication history OR AB medication history OR TI therapy management OR AB therapy management OR TI follow-up OR AB follow-up OR TI follow up OR AB follow up) | MH/TI/AB |
| **Context** | **Sub-terms** | **Search options** |
| **Emergency** | MH "Emergency Services, Hospital+" OR TI Emergency OR AB Emergency | MH/TI/AB |

**Search strategy: 1341 results**

((MH "Pharmacists+" OR MH "Pharmacy Technicians+" OR MH "Pharmacy Students+" OR MH "Pharmacy Residencies+" OR MH "Pharmacy+" OR MH "Pharmaceutical Services+" OR TI Pharmacist* OR AB Pharmacist* OR TI Pharmacy OR AB Pharmacy) AND (MH "Continuity of Patient Care+" OR MH "Medication Reconciliation+" OR MH "Medication Review+" OR MH "Medication Therapy Management+" OR MH "Patient Education as Topic+" OR MH "Counseling+" OR TI Transitions of care OR AB Transitions of care OR TI transition of care OR AB transition of care OR TI transition-of-care OR AB transition-of-care OR TI transitions-of-care OR AB transitions-of-care OR TI TOC OR AB TOC OR TI transitional care OR AB transitional care OR TI Care Transition* OR AB Care Transition* OR TI hospital to home OR AB hospital to home OR TI Discharge OR AB Discharge OR TI Transfer OR AB Transfer OR TI Admission OR AB Admission OR TI Coordination OR AB Coordination OR TI Continuity of Care OR AB Continuity of Care OR TI Care Continuity OR AB Care Continuity OR TI Handoff OR AB Handoff OR TI Hand off* OR AB Hand off* OR TI Hand over OR AB Hand over OR TI Handover OR AB Handover OR TI Hands off OR AB Hands off OR TI Education OR AB Education OR TI Counseling OR AB Counseling OR TI medication chart OR AB medication chart OR TI Review OR AB Review OR TI Reconcil* OR AB Reconcil* OR TI medication history OR AB medication history OR TI therapy management OR AB therapy management OR TI follow-up OR AB follow-up OR TI follow up OR AB follow up) AND (MH "Emergency Services, Hospital+" OR TI Emergency OR AB Emergency))

***Table S3:*** *Search strategy for Embase*

| **No.** | **Query** | **Results** | **Date** |
| --- | --- | --- | --- |
| #40 | ('clinical pharmacy'/exp OR 'hospital pharmacy'/exp OR 'clinical pharmacist'/exp OR 'pharmacist'/exp OR 'pharmacy student'/exp OR 'pharmacy technician'/exp OR 'pharmacy (shop)'/exp OR pharmacy:ti,ab OR pharmacist*:ti,ab) AND ('transitional care'/exp OR 'hospital discharge'/exp OR 'clinical handover'/exp OR 'patient transport'/exp OR 'medication therapy management'/exp OR 'patient education'/exp OR 'patient counseling'/exp OR 'transition* of care':ab,ti OR 'care transition*':ab,ti OR 'transitional care':ab,ti OR 'continuity of care':ab,ti OR coordination:ab,ti OR 'care continuity':ab,ti OR handoff*:ab,ti OR 'hand over':ab,ti OR handover:ab,ti OR 'hand off':ab,ti OR reconcil*:ab,ti OR review:ab,ti OR 'medication history':ab,ti OR 'follow up':ab,ti OR education:ab,ti OR counseling:ab,ti) AND ('emergency health service'/exp OR 'emergency ward'/exp OR 'hospital emergency service'/exp OR emergency:ab,ti) | 5501 | 11 Dec 2024 |
| #39 | 'emergency health service'/exp OR 'emergency ward'/exp OR 'hospital emergency service'/exp OR emergency:ab,ti | 671292 | 11 Dec 2024 |
| #38 | 'transitional care'/exp OR 'hospital discharge'/exp OR 'clinical handover'/exp OR 'patient transport'/exp OR 'medication therapy management'/exp OR 'patient education'/exp OR 'patient counseling'/exp OR 'transition* of care':ab,ti OR 'care transition*':ab,ti OR 'transitional care':ab,ti OR 'continuity of care':ab,ti OR coordination:ab,ti OR 'care continuity':ab,ti OR handoff*:ab,ti OR 'hand over':ab,ti OR handover:ab,ti OR 'hand off':ab,ti OR reconcil*:ab,ti OR review:ab,ti OR 'medication history':ab,ti OR 'follow up':ab,ti OR education:ab,ti OR counseling:ab,ti | 6056577 | 11 Dec 2024 |
| #37 | 'clinical pharmacy'/exp OR 'hospital pharmacy'/exp OR 'clinical pharmacist'/exp OR 'pharmacist'/exp OR 'pharmacy student'/exp OR 'pharmacy technician'/exp OR 'pharmacy (shop)'/exp OR pharmacy:ti,ab OR pharmacist*:ti,ab | 245490 | 11 Dec 2024 |
| #36 | emergency:ab,ti | 551217 | 11 Dec 2024 |
| #35 | 'hospital emergency service'/exp | 11386 | 11 Dec 2024 |
| #34 | 'emergency ward'/exp | 239038 | 11 Dec 2024 |
| #33 | 'emergency health service'/exp | 361662 | 11 Dec 2024 |
| #32 | counseling:ab,ti | 128595 | 11 Dec 2024 |
| #31 | education:ab,ti | 843895 | 11 Dec 2024 |
| #30 | 'follow up':ab,ti | 2103727 | 11 Dec 2024 |
| #29 | 'medication history':ab,ti | 4383 | 11 Dec 2024 |
| #28 | review:ab,ti | 2944080 | 11 Dec 2024 |
| #27 | reconcil*:ab,ti | 28837 | 11 Dec 2024 |
| #26 | 'hand off':ab,ti | 707 | 11 Dec 2024 |
| #25 | handover:ab,ti | 3846 | 11 Dec 2024 |
| #24 | 'hand over':ab,ti | 1158 | 11 Dec 2024 |
| #23 | handoff*:ab,ti | 3528 | 11 Dec 2024 |
| #22 | 'care continuity':ab,ti | 1101 | 11 Dec 2024 |
| #21 | coordination:ab,ti | 162540 | 11 Dec 2024 |
| #20 | 'continuity of care':ab,ti | 13244 | 11 Dec 2024 |
| #19 | 'transitional care':ab,ti | 3381 | 11 Dec 2024 |
| #18 | 'care transition*':ab,ti | 4118 | 11 Dec 2024 |
| #17 | 'transition* of care':ab,ti | 4746 | 11 Dec 2024 |
| #16 | 'patient counseling'/exp | 54714 | 11 Dec 2024 |
| #15 | 'patient education'/exp | 130868 | 11 Dec 2024 |
| #14 | 'medication therapy management'/exp | 16527 | 11 Dec 2024 |
| #13 | 'patient transport'/exp | 37647 | 11 Dec 2024 |
| #12 | 'clinical handover'/exp | 11960 | 11 Dec 2024 |
| #11 | 'hospital discharge'/exp | 203983 | 11 Dec 2024 |
| #10 | 'transitional care'/exp | 6410 | 11 Dec 2024 |
| #9 | pharmacist*:ti,ab | 99244 | 11 Dec 2024 |
| #8 | pharmacy:ti,ab | 111963 | 11 Dec 2024 |
| #7 | 'pharmacy (shop)'/exp | 105805 | 11 Dec 2024 |
| #6 | 'pharmacy technician'/exp | 3128 | 11 Dec 2024 |
| #5 | 'pharmacy student'/exp | 10469 | 11 Dec 2024 |
| #4 | 'pharmacist'/exp | 106737 | 11 Dec 2024 |
| #3 | 'clinical pharmacist'/exp | 4646 | 11 Dec 2024 |
| #2 | 'hospital pharmacy'/exp | 17165 | 11 Dec 2024 |
| #1 | 'clinical pharmacy'/exp | 12486 | 11 Dec 2024 |

***Table S4:*** *Data Extraction Tool – Part A (Overall Characteristics)*

| **First Author** | **Publication Year** | **Country** | **Study Design** | **Total Sample Size** | **Study Duration (days)** | **Study Follow-up Period (days)** | **Inclusion Criteria (Targeted Population)** | **Justification for Inclusion Criteria** | **General Description of Intervention** | **Description of Comparison (If Applicable)** | **Outcome Descriptions** | **Quality Measure Category – Primary Outcomes** | **Quality Measure Category – Secondary Outcomes** | **Overall effect of the intervention (Favors Intervention, Favors Control, Neutral) – Primary Outcome** |
| --- | --- | --- | --- | --- | --- | --- | --- | --- | --- | --- | --- | --- | --- | --- |
|  |  |  |  |  |  |  |  |  |  |  |  |  |  |  |
|  |  |  |  |  |  |  |  |  |  |  |  |  |  |  |
|  |  |  |  |  |  |  |  |  |  |  |  |  |  |  |
|  |  |  |  |  |  |  |  |  |  |  |  |  |  |  |

***Table S5:*** *Data Extraction Tool – Part B ( Reporting of TiDieR Items & Intervention Design), adapted from Hoffman et al. [1]*

| **First Author, Year** | **Intervention Development** | **Item 1. Brief name** | **Item 2. Why** | **Item 3. Materials** | **Item 4.Procedures** | **Item 5. Who provided** | **Item 6. How** | **Item 7. Where** | **Item 8. When and how much** | **Item 9. Tailoring** | **Item 10. Modifications** | **Item 11. How well (planned)** | **Item 12: How well (actual)** |
| --- | --- | --- | --- | --- | --- | --- | --- | --- | --- | --- | --- | --- | --- |
|  |  |  |  |  |  |  |  |  |  |  |  |  |  |
|  |  |  |  |  |  |  |  |  |  |  |  |  |  |
|  |  |  |  |  |  |  |  |  |  |  |  |  |  |
|  |  |  |  |  |  |  |  |  |  |  |  |  |  |

***Table S6:*** *Showing the summary of the TIDieR items (1-6) for the pharmacy-supported transitional care interventions in the emergency department*

|  |  |  |  |  |  |  |
| --- | --- | --- | --- | --- | --- | --- |
| First author,  Publication year | **Item 1. Brief name** | **Item 2. Why** | **Item 3. Materials** | **Item 4.**  **Procedures** | **Item 5. Who provided** | **Item 6. How** |
| Kaucher, 2025 [2] | ED pharmacist-led nPEP counseling for sexual assault survivors | Evidence gap, Opportunity for pharmacists, Supporting evidence, Unique ED opportunities/ challenges, Cost, Equity and access to care | • EHR • Telephone  • Follow-up survey • 28-day supply of nPEP | 1. 28-day supply of nPEP free of charge was provided to the patient at ED discharge. 2. Counseling by EM clinical pharmacists 3. Referral to STD clinic. | EM clinical pharmacists: trained in the management of sexual assault pharmacotherapy and public health measures for at-risk patients | • Face-to-face in the clinic with telephone follow-up |
| Boot, 2024 [3] | Pharmacy-led ED culture callback program | Evidence gap, Opportunity for pharmacists, Supporting evidence, Unique ED opportunities/ challenges | • EHR • Ascension guidelines and BSIs based on site-specific guidelines • Printed culture reports • Letters | 1. Chart review was performed by the pharmacist 2. Recommendation to therapy was sent to GP for approval 3. Patients were contacted for any change in prescriptions or in case of positive STI results to provide counseling | (trained by an ED pharmacist)  Seven clinical pharmacists, including four ED pharmacists, one PGY2-EM pharmacy resident, and two critical care pharmacists | • Patient follow-up through telephone/ letter |
| Reilly, 2024 [4] | Geriatric emergency department Intervention pharmacist service | Opportunity for pharmacists, Supporting evidence, Unique ED opportunities/ challenges, Cost | • STOPP/START criteria. • Telephone & Email | 1. The GEDI pharmacist works as part of the MDT to provide care for older patients. 2. Role included: medication review, assessment of medication adherence, medicines reconciliation, and the application of the STOPP/START criteria | Geriatric Emergency Department Intervention (GEDI) pharmacist | • Face-to-face  • Telephone calls by GEDI nurses  • Recommendations sent to GPs via "secure electronic communication" |
| Lee, 2024 [5] | Partnered pharmacist discharge prescription planning (PPDPP) | Evidence gap, Opportunity for pharmacists, Supporting evidence, Unique ED opportunities/ challenges | • EHR | 1. Medication management decision and planned discharge prescriptions were discussed by the pharmacist and medical officer | ED pharmacists: Pharmacists were required to satisfactorily complete a ward-based assessment using the Society of Hospital Pharmacists of Australia mini-clinical evaluation exercise to provide the intervention. | • Face-to-face discussion with MO, with documentation done in the EHR |
| Selman, 2024 [6] | ED pharmacist led high-risk drugs' deprescribing for older adults | Evidence gap, Unique ED opportunities/ challenges | •EHR • STEADI-Rx initiative • 2019 AGS Beers criteria • Fax | 1. Medication reconciliation was performed by EMP 2. High-risk medications were identified, and education to patients and care partners was provided. 3. Recommendations were communicated through patients’ EHR | ED pharmacists | • Electronically through EHR, and face-to-face patient interview and education.  • The recommendations were communicated through fax or EHR to the PCPs |
| Martínez, 2024 [7] | Clinical pharmacists' integration into the ED discharge process | Evidence gap, Opportunity for pharmacists, Unique ED opportunities/ challenges, Cost, Equity and access to care | • EHR • Telephone • Written educational material | 1. Recommendations for optimal pharmacotherapy were made by the CP. 2. Education to patients and their caregivers on the therapeutic plan to be followed in the ED and at discharge. | Clinical pharmacist | • The intervention was delivered at ED discharge with a phone follow-up by a blinded independent pharmacist. • Verbal and written education provided to patients and caregivers |
| Maleki, 2024 [8] | Pharmacist-led review of high needs patients in the ED | Evidence gap, Opportunity for pharmacists, Supporting evidence, Unique ED opportunities/ challenges, Cost, Policy | • High Needs Patients Identification tool • EHR | 1. High Needs Patients had BPMH completed by EMP. 2. Medication related problems and errors were Identified and discussed with the ED and/or admitting unit medical staff. | ED pharmacist | Not reported |
| Sofeso, 2024 [9] | Prospective pharmacist review of discharge antibiotics for urinary tract infections in the ED | Evidence gap, Opportunity for pharmacists, Supporting evidence, Unique ED opportunities/ challenges | • ED-specific algorithm for UTIs  • A documentation tool within the EHR • EHR | 1. Orders were prospectively reviewed based on adherence to local guidelines by EMP. 2. Medication related issues were identified, and recommendations were addressed to clinicians. | EM pharmacists | • A documentation tool within the EHR was created for EMP to document their interventions and recommendations to EM clinicians |
| Tran-Nguyen, 2024 [10] | Preventing readmissions in older adults: a pharmacist-led ED collaborative | Evidence gap, Opportunity for pharmacists, Cost | • EHR • Comprehensive medication list titled “Pharmacy Progress Note" | - Comprehensive medication management consultations, discharge liaison services, and other pharmacy related interventions were provided to eligible participants by the pharmacist.  1. Follow up with the patient’s local pharmacy to communicate therapy changes if needed. | Pharmacist | The intervention was delivered in the ED with electronic review, medication reconciliation, and patient education |
| Atey, 2024 [11] | Partnered Pharmacist Medication Charting (PPMC) | Evidence gap, Opportunity for pharmacists, Supporting evidence, Unique ED opportunities/ challenges | • Local THS-specific list of TCMs  • EHRs. • Shared medication treatment plan (SMTP). • THS-specific VTE risk assessment form. • National Inpatient Medication Chart (NIMC) | - BPMH and clinical review were conducted by EMP. - SMTP was co-developed with a medical officer - Medication reconciliation was conducted by ward pharmacist | ED pharmacist, ward pharmacist | The best possible medication history (BPMH) was obtained through structured patient interviews and secondary sources, such as caregivers, EHRs, and community pharmacies. |
| Wang, 2024 [12] | A pharmacist-driven deprescribing protocol for negative urine and sexually transmitted infection cultures | Evidence gap, Opportunity for pharmacists, Supporting evidence, Unique ED opportunities/ challenges | • Daily culture reports by BD MedMined surveillance data  • Institution antibiogram and clinical guidelines • Standard script when contacting patients to determine symptoms • Telephone. | - De-escalation or potentially stopping antibiotics for patients with a negative culture was evaluated by pharmacy residents - Follow-up plans during patient outreach and counseling were provided. | Residency-trained, board-certified ED pharmacists (certified critical care pharmacists) -Postgraduate year 1 (PGY1) pharmacy residents under supervision of ED pharmacist and ED physician | • Phone call  • Postal Service mail. |
| Kofoed, 2023 [13] | Pharmacist-driven follow-up results  (FUR) process | Opportunity for pharmacists | • EHR  • DMAIC (Define, Measure, Analyze, Improve, Control), a Six Sigma methodology. • telephone | - **Upon** a negative urine culture result, a message is sent to the pharmacist’s inbox to prompt further review of the patient’s record and disposition.  1. Patients were contacted, and the pharmacist then canceled the prescription in the patient’s medical record and forwarded the cancellation to a designated provider. | Pharmacist group,  recently started a residency program | Through phone |
| Pham, 2023 [14] | Pharmacist-led, urinary culture follow-up after discharge from the emergency department | Evidence gap, Opportunity for pharmacists, Supporting evidence, Unique ED opportunities/ challenges | • CPA protocol • EHR | 1. ED pharmacists were privileged to independently adjust (add, adjust, and discontinue) antibiotic regimens based on urine culture results for antibiotics within the specifications of the protocol. 2. Patients were contacted for counseling/education If an intervention was required | ED pharmacists | EHR review and documentation Patient Interview  Intervention was done on individual basis |
| Jovevski, 2023 [15] | Ed pharmacist-led med reconciliation & deprescribing for high-risk seniors | Evidence gap, Opportunity for pharmacists, Supporting evidence, Unique ED opportunities/ challenges, Policy | • EHR • Standardized note template • 2019 American Geriatrics Society Beers Criteria | 1. Standardized process to screen for high-risk older adults and identify PIMs based on guidelines and using clinical judgment by pharmacists. 2. Recommendations were communicated to PCP through a standardized note template within the EHR. | Two board-certified pharmacotherapy specialist pharmacists | EHR-based notification alert,  and a standardized note template |
| Andrade, 2023 [16] | Combined collaborative drug therapy management (CDTM) and the rapid administration of intravenous antimicrobials by an infectious diseases specialist (RAIDS) Program for MDR Management in the ED. | Evidence gap, Opportunity for pharmacists, Supporting evidence, Policy | • Education materials: practice cases and treatment algorithms, and sample dialog scripts of patient interaction • Two treatment guidelines. • The ED Culture Callback Collaborative Drug Therapy Management (CDTM) protocol • Rapid Administration of Intravenous Antimicrobials by an Infectious Disease Specialist (RAIDS) protocol • Telephone | 1. Positive MDR microbiology culture results for ED discharged patients were relayed to EMPs 2. Treatment appropriateness and adjustment was communicated with patients as needed without physician notification. | EM pharmacist, PGY2 EM resident, PGY1 pharmacy residents, Education provided to involved personnel, PGY1 competency checked by EM Pharmacotherapy Specialist | • Through chart review  • EMPs contact the patient by telephone and/or letter when necessary |
| Dunn, 2023 [17] | Pharmacist-led telephone outreach program post-ED discharge | Evidence gap, Opportunity for pharmacists, Supporting evidence, Unique ED opportunities/ challenges, Cost, Equity and access to care | • Telephone  • Fax | - Medication reconciliation interview with the subject or caregiver via telephone 48–96 hours after ED discharge.  1. Discrepancies were communicated to the primary provider via fax or telephone. | Pharmacists | • The intervention was delivered through telephone consultation The intervention was done on an individual basis |
| Atey, 2023 [18,19,20] | Partnered pharmacist medication  charting (PPMC) | Evidence gap, Opportunity for pharmacists, Supporting evidence, Unique ED opportunities/ challenges, Cost | PPMC-credentialing pathway requirements for ED pharmacists:  • Clinical assessment via the Society of Hospital Pharmacists of Australia (SHPA)’s Clinical Competency Achievement Tool (ClinCAT). • Objective structured clinical examination (OSCE)-credentialing assessment | 1. BPMH and clinical review were conducted by EMP. 2. SMTP was co-developed with a medical officer 3. Medication reconciliation was conducted by ward pharmacist | Over 20 ED pharmacists trained and credentialled to provide Partnered pharmacist medication charting (PPMC). | • Structured patient  interviews • Medication chart |
| Zhao, 2023 [21] | Emergency department clinical pharmacist integration within a discharge urine culture follow-up program | Opportunity for pharmacists, Supporting evidence, Unique ED opportunities/ challenges, Policy | • Urine culture review via EHR | 1. Positive urine cultures are flagged in EHR for review by pharmacists. 2. Treatment recommendations are discussed with the provider to determine the final treatment plan. | ED clinical pharmacist | Not reported |
| Benson, 2023 [22] | Pharmacist-led urine culture follow-ups | Evidence gap, Opportunity for pharmacists, Supporting evidence, Unique ED opportunities/ challenges, Equity and access to care | • EHR | - A chart review for each urine culture is conducted to determine whether intervention or modification wererequired.  1. If action was required, the pharmacist consulted an ED provider for an agreement and then documented the encounter. | Pharmacists | Hospital’s EHR documentation |
| Ng, 2022 [23] | ED SSU smoking cessation service | Evidence gap, Opportunity for pharmacists, Supporting evidence, Unique ED opportunities/ challenges | • Breath CO levels (Smokerlyzer test) | - ED smoking cessation service included a bedside counseling session by a pharmacist. - Pharmacists provide an individualized quit plan. - Education on the harmful effects of smoking, benefits of quitting, and ways to cope with withdrawal symptoms. | Trained pharmacists certified by the Singapore Health Promotion Board as smoking cessation counselors | Face-to-face |
| Nymoen, 2022 [24] | Systematic medication review in emergency department | Evidence gap, Opportunity for pharmacists, Supporting evidence, Unique ED opportunities/ challenges | • Medication reconciliation checklist • Electronic prescription database • Validated medication review-tool • Computer resources (e.g., interaction databases, summary of product characteristics for drugs, and medical databases) • EHR | - Medication reconciliation and identification of predefined DRP using predefined DRP categories.  1. Recommendations were suggested by pharmacists and documented in the EHR and orally communicated to the ED physician. | Experienced clinical pharmacists (study pharmacists). To standardize study procedures, written operation procedures were developed. All involved study pharmacists were familiar with these operational procedures before study start. | • EHR • Oral communication with patients and the in-charge ED physician |
| Ogilvie, 2022 [25] | A collaborative pharmacist prescribing model in ED | Evidence gap, Opportunity for pharmacists, Supporting evidence, Policy | • Progress notes • National Inpatient Medication Chart (NIMC) • Password-protected Microsoft Excel™ | - Collaborative plan for optimizing the medications whilst the patient is still in ED. | Two pharmacists. One pharmacist (primary author) was the credentialled extended scope pharmacist prescriber who remained in the same role throughout the study, but the second ED pharmacist had a rotational role | EHR |
| Stewart, 2021 [26] | Pharmacist-led Transitional Care Clinic (TCC) for hypertension management | Evidence gap, Opportunity for pharmacists, Supporting evidence, Unique ED opportunities/ challenges, Policy, Equity and access to care | • The Joint National Committee Guidelines for Management of Hypertension. • REDCap electronic data capture tools hosted at Wayne State University • EHR | - Comprehensive hypertension management under a CPA lead by the pharmacist.  1. At each visit, pharmacists could initiate/modify therapy, provide lifestyle counseling, conduct BP/lipid monitoring, address access barriers, and document in the EHR. | One clinical pharmacist, student pharmacists  and pharmacy residents | TCC clinic visits were documented in the DMC EHR. |
| Lineberry, 2021 [27] | Pharmacist-targeted discharge prescription review in an emergency department | Evidence gap, Unique ED opportunities/ challenges | • EHR  • Medication list • Standard drug information resources, internal and external guidelines (no names) | - Signed discharge prescription on the targeted prescription list alerts were sent to EMPs - Prescriptions were reviewed for potential interventions | EM Pharmacist | Electronic review with contacting patients/prescribers for changes as needed |
| Celikkayalar, 2021 [28] | Collaborative medication reviews (CMR) led by a clinical pharmacist | Evidence gap, Supporting evidence, Unique ED opportunities/, Policy | • A paper form for CMR documentation and communication. • Electronic evidence-based medication risk management databases for identifying IP events. • RENBASE™, a decision support database for information on the safe and effective use of drugs in patients with renal failure  • The Finnish List of Potentially Inappropriate Medication for Older Persons Population (Meds75+) • The Finnish Current Care Guidelines • Package Leaflets | - Documented prescribed pre-admission medications were reviewed by an ED pharmacist. - Appropriateness of prescribing was discussed with physicians, and confirmed changes to medications were documented in the EHR. | Specially trained ED pharmacist | • A paper form for CMR documentation and communication • Confirmed changes to medications were documented to medication records |
| Castillo, 2021 [29] | Discharge prescription optimization by emergency medicine pharmacists | Evidence gap, Opportunity for pharmacists, Unique ED opportunities/, Policy | • Computerized physician order entry. • EHR • Patient identification tool | - ED discharge prescriptions were generated by prescribers using CPOE within the patient’s EHR.  1. Prescriptions and interventions were discussed and documented. | A core team of four EM pharmacists who provided  dedicated clinical services | EHR |
| Rainess,2021 [30] | Addition of a clinical pharmacist service to a midlevel provider-driven culture follow-up program | Evidence gap, Supporting evidence, Unique ED opportunities/, Policy | • Infectious Diseases Society of America (IDSA) guideline • Culture results • The hospital’s antibiogram • Medication packages insert • Online databases such as Lexicomp | - Callback culture list is reviewed daily by CP, and recommendations were made based on results, susceptibility, and guidelines. - Recommendations were forwarded to the provider for approval. | Multiple residency trained clinical pharmacist, Three were board certified | Further clarification of pharmacists' recommendations was done through electronic communications |
| Olson, 2020 [31] | Pharmacist-initiated culture review process | Opportunity for pharmacists, Supporting evidence, Unique ED opportunities/ challenges | • Messaging system for communication between healthcare providers • Telephone • Letter | - Final results from urine and STI cultures were reviewed by pharmacists. - Written recommendations based on susceptibility or patient factors (initiation, modification, or discontinuation of antibiotics) are sent to advanced practice providers. | ED pharmacist | • The cultures are electronically reviewed with the recommendations sent to advanced practice providers (APPs) through an inbox messaging system.  • Patients are contacted by APPs through phone or letter. |
| Roels, 2020 [32] | Collaborative Practice Agreement (CPA) | Evidence gap, Opportunity for pharmacists, Supporting evidence, Policy | • EHR  • Paper tracking sheet • Telephone • Letter | - The ED clinical pharmacist reviewed the patient’s case, microbiology results, and discharged medications to determine the appropriate clinical action. - If supported by the CPA, the pharmacist intervened independently. | ED clinical pharmacists | Not reported |
| Lim, 2020 [33] | Rivaroxaban after-hours pack for outpatient deep vein thrombosis management | Unique ED opportunities/ challenges | • Paper-based patient registry • GuidanceMS database • Telephone  • Rivaroxaban after-hours pack: 3-day supply of Rivaroxaban 15 mg (six tablets), patient product information booklet, and emergency contact details. | - After-hours pack is given to patients (3-weeks supply of rivaroxaban) who meet the criteria. - Follow up phone calls and clot clinic referrals by pharmacists. | ED Pharmacist | • The intervention was provided in the ED based on patients' information from the patient registry and the decision support tool 'GuidanceMs'.  • Pharmacists contacted patients through phone, communicated patient details, and identified issues through Email.  • Patients were also followed up with face-to-face appointments in the Clot Clinic. |
| Tweedle, 2020 [34] | Review of positive cultures by clinical pharmacists | Evidence gap, Opportunity for pharmacists, Supporting evidence, Policy | • Medical record • Interpreter  • Letters  • Telephone | - Positive culture results were reviewed to ensure appropriate antibiotics and recommendations were documented. - Interventions were all done via telephone and letters. | Board-certified, PGY1 residency-trained ED clinical pharmacists | Telephone and letter |
| Wu, 2020 [35] | Health system-wide, pharmacist-driven emergency department laboratory follow-up and antimicrobial management program | Evidence gap, Opportunity for pharmacists, Supporting evidence, Unique ED opportunities/ challenges | • Therapeutic guides • Letter • Telephone | - Lab results were reviewed and discussed daily. - If necessary, pharmacists called in or electronically prescribed a new prescription on behalf of the clinician. | ED and ID pharmacists. Program training was provided to all participating pharmacists via online, live education sessions and on-site training by project leads. | • Pharmacists called in or electronically prescribed a new prescription on behalf of the clinician.  • Patients were contacted by telephone and letter |
| Houlind, 2020 [36] | A collaborative medication review and deprescribing intervention in an ED | Evidence gap, Opportunity for pharmacists, Supporting evidence, Unique ED opportunities/ challenges | • Shared Medication Card (SMC)  • EHR • Medication Review Tools: (STOPP) criteria, a drug–drug interaction database (SFINX), and Renbase® (renal dosing database) • National prescribing guidelines | - An initial medication reconciliation upon admission. - A collaborative medication review intervention focused on deprescribing was sent to geriatricians for review. - A final ready-to-discharge medication reconciliation. | Senior clinical pharmacist | The Medication reconciliation, medication review, and deprescribing were done based on information from a central database (Shared Medication Card), the patients' EHR, national guidelines, and deprescribing tools |
| Layman, 2020 [37] | Clinical pharmacist-led transitional clinic with post-discharge face-to-face and telephone follow-up within 2 weeks | Opportunity for pharmacists, Supporting evidence, Cost | • Adherence and educational materials (blood pressure monitors, medication planners, pill splitters, and scales) • Telephone | - Pharmacist-led TCC using CPSs to independently manage medications, perform assessments, order labs, and provide disease state education via face-to-face or phone within 2 weeks post-discharge. | Clinical pharmacy specialists, independent midlevel practitioners under a scope of practice | The post-discharge follow-up was delivered face-to-face in the clinic or through phone/video conferencing for rural patients with limited access to care. |
| Pearson, 2020 [38] | Post ED discharge telephonic outreach and assessment by a clinical pharmacist, with triaging to other staff if necessary | Evidence gap, Supporting evidence, Cost | • Telephone • EHR  • American Geriatrics Society Beers Criteria® | - Comprehensive problem-based therapeutic assessments were developed, and patients were contacted for medication reconciliation and education. | Three clinical pharmacists | • EHR  • Patient outreach through telephone |
| Kitchen, 2020 [39] | Medication review delivered by pharmacists to ED patients | Opportunity for pharmacists, Supporting evidence | • Hospital charts • The hospitals’ electronic patient information system • Telephone • Fax • Written Notes | - Examination of ED patient medications which included obtaining BPMH, optimizing medications, and identifying and addressing medication-related problems and adverse drug events. - The recommendations were communicated to physicians. | Residency-trained clinical pharmacists with a minimum of 2 years’ working experience in an acute care hospital. They were oriented in a 2-week training and pilot period (from the protocol) | Patients' charts, phone calls, or tax letters. |
| Shealy, 2020 [40] | Pharmacist-Driven Culture and STI Testing Follow-Up Program in the Emergency Department | Evidence gap, Opportunity for pharmacists, Supporting evidence, Unique ED opportunities/ challenges, Policy | • A live alert system for positive cultures and STI updates integrated into the decision support tools • Local and national antimicrobial prescribing/ clinical management guidelines | - PGY1 resident review and triage culture and STI RDT results daily for ED patients. - Communication of the proposed recommendation to the ED physician and implementation of the plan. | PGY1 resident with AMS training | • Electronically • Follow up (by phone or mail) |
| Loborec, 2020 [41] | Privileging pharmacists to independently manage the ED discharge microbiologic test result review process | Evidence gap, Opportunity for pharmacists, Supporting evidence, Policy | • EHR  Pharmacist prerequisite training for the intervention • SPP workflow training • SPP Antimicrobial Tx training • SPP case-based exam | - SPP Reviewing microbiologic results and contacting patients - Act on the results if an intervention is needed, with the pharmacist adding prescriptions on behalf of the prescriber. | Specialty practice pharmacists (SPPs), completed PGY2 pharmacy residency training, with 5 years of experience practicing in ED and 4 years facilitating ED microbiological test results follow-up. Moreover, the pharmacist underwent competency training | • Electronic review.  • Pharmacists communicated any changes to patients. However, the mode of contact was not mentioned in the article. |
| Eswaran, 2020 [42] | ED-based take-home naloxone (THN) program | Evidence gap, Supporting evidence, Policy | • A free “THN” kit  • Custom electronic medical record order was created for dispensing of THN kits • 2 educational handouts provided to patients | - Take-home naloxone kit is dispensed to selective patients. - The ED pharmacist then instructs the patient on how to recognize opioid overdose and the proper use of the naloxone kit before discharge. | ED clinical pharmacists who completed an online training module "Prescribe-to-Prevent", took a posttest, and received 1.5 hours of continuing education credit | Face-to-face |
| Giruzzi, 2020 [43] | Antimicrobial Stewardship pharmacist culture review service | Evidence gap, Supporting evidence, Unique ED opportunities/ challenges, Policy | • Infection prevention database for positive cultures collected in the ED • EHRs • Infectious Diseases Society of America (IDSA) guidelines | - ASP pharmacists evaluated ED cultures during their daily antimicrobial stewardship activities and evaluated prescribed regimen for adequacy. - If therapeutic changes were made, the patients were contacted. | Three antimicrobial stewardship (ASP) pharmacists, completed an antimicrobial stewardship certification program and at least 1 year of residency training | EHR |
| Cabilan, 2019 [44] | Pharmacist-led discharge medication counselling | Evidence gap, Opportunity for pharmacists, Supporting evidence, Unique ED opportunities/ challenges, Policy | • Consumer Medication Information (CMI) leaflet • Patient prescription and medications  • Patient satisfaction using SIMS (Satisfaction with Information about Medicines Scale) | - Pharmacists provided bedside consultation prior to discharge and provided CMI leaflets. | Pharmacist, were briefed about the intervention prior to working in the ED by the investigators | Face-to-face |
| Schwab, 2019 [45] | Multidisciplinary anticoagulation initiative in the ED | Evidence gap | • Risk scoring tools (CHA2DS2-VASc and HAS-BLED) • Telephone  • Patients' medical records | - MDT assessed patients for oral anticoagulation based on CHA2DS2-VASc and HAS-BLED score. - Treatment plans, follow-up appointments, and TOC services were provided. | ED pharmacist | Telephone |
| Fay, 2019 [46] | Pharmacist-led antimicrobial stewardship program (ASP) in urgent care. | Evidence gap, Unique ED opportunities/ challenges, Policy | • Orientation to the hospital’s outpatient antibiograms and outpatient empiric therapy guidelines. • Telephone • EHR | - Pharmacists independently review microbiology results and contact patients for follow-up assessment and intervention per CPA. | The ID and ED pharmacists, PGY1 pharmacy residents, 4th year Pharm.D. students | • Electronic review  • Telephone follow-up |
| Southerland, 2011 [47] | Inter-professional transitions of care services for older adults discharged from the ED | Opportunity for pharmacists, Supporting evidence, Unique ED opportunities/ challenges, Cost | • Patients’ intake form and discharge summary • Community pharmacy records  • Telephone | - Two TOC services; pharmacy-only or pharmacy and home health care. - Medication reconciliation, a prospective drug utilization review, CMR, and nurses’ home visits. | Community pharmacists | Telephone |
| Lacy, 2018 [48] | A pharmacy-driven post-emergency department (ED) transitions of care program | Evidence gap, Supporting evidence, Unique ED opportunities/ challenges, Cost | • Telephone • EHR  • Call script and an electronic template for post-ED discharge follow-up calls | - Phone calls by pharmacists within 7 days of ED discharge to perform medication reconciliation using standardized script. | PGY1 pharmacy resident or fourth year pharmacy students under the supervision of clinic-based pharmacists | • Telephone |
| Santolaya-Perrín, 2018 [49, 56] | Multidisciplinary intervention for medication review in elderly ED patients | Evidence gap, Opportunity for pharmacists, Unique ED opportunities/ challenges | • STOPP-START criteria • Medications list • Emergency discharge report • EHR • Email | - Pharmacists review the chronic medications of the patients and identify any PIPs according to the STOPP-START criteria. - Recommendations to modify the treatment is sent to the PCP. | Pharmacist | The intervention was delivered during the ED stay of the patient, with communication with the other providers being done by Email |
| Chu, 2017 [50] | A rivaroxaban program for acute venous thromboembolism upon emergency department discharge | Cost Saving/ Economic Impact | • Rivaroxaban Starter Pack™  • Telephone | - ED pharmacists reviewed past medical history, home medication, and major interactions; and advised prescribers on initial dosing of the Rivaroxaban Starter Pack™. - Patients were counseled on the pack, and follow-up phone calls were conducted by pharmacists. | ED pharmacist | • Face-to-face counselling  • Telephone follow-up |
| DiRenzo, 2017 [51] | Outpatient treatment of VTE with rivaroxaban in the pharmacist-managed clinic | Opportunity for pharmacists, Policy | • EHR • Assessment of adherence using • The four-item Modified Morisky Adherence Scale. • CHEST guidelines. | - The clot clinic pharmacist can modify rivaroxaban, warfarin, and enoxaparin prescriptions if originally prescribed by a physician. - Patients are counseled on medication use, adherence, side effects, drug interactions, lifestyle modifications, smoking cessation, and other issues as they arise. | 1- 2 clinical pharmacists (internal medicine and critical care) | • HER review  • Face-to-face counselling |
| Hohl, 2017 [52] | Clinical pharmacist led systematic medication review | Evidence gap, Opportunity for pharmacists, Unique ED opportunities/ challenges, Policy, Cost | • Norwegian patient registry  • EHR • Interaction databases, summary of product characteristics for drugs, and medical databases | - Systematic medication review including medication reconciliation conducted by pharmacist during the ED stay. | Experienced clinical pharmacists, study specific operation procedures developed and shared with implementers | • Face-to-face |
| Zdyb, 2017 [53] | Anticoagulation discharge education provided by ED pharmacists | Evidence gap, Opportunity for pharmacists, Supporting evidence, Unique ED opportunities/ challenges, Cost | • EHR  • A standardized electronic form for pharmacists (bedside documentation, callback documentation) • Telephone | - Patients were flagged for pharmacist counseling using a form upon anticoagulant order. - Pharmacists also assisted with insurance verification and affordability analysis on physician requests. | ED pharmacist | • Face-to-face counselling • Telephone follow-up |
| Hohner, 2016 [54] | Emergency department (ED)–based clinical pharmacist transitions-of-care (TOC) program | Evidence gap, Opportunity for pharmacists, Supporting evidence, Unique ED opportunities/ challenges, Equity and access to care | • A standardized form specific to the underlying disease  • Referral form to the home visit program coordinator • EHR | - The pharmacist reviewed current home medications, assessed medication compliance or administration techniques, and addressed any patient-specific concerns regarding medications using a form. - Modification of the care plan as appropriate and scheduling follow-up appointments. | ED clinical pharmacy specialists, a PGY2 emergency medicine pharmacy resident, an ambulatory care clinical pharmacy specialist, and a PGY2 ambulatory care pharmacy resident | • Face-to-face |
| Lingenfelter, 2016 [55] | ED pharmacist urine culture review | Opportunity for pharmacists, Unique ED opportunities/ challenges | • EHR  • Urine culture assays • Telephone | - ED pharmacists review all cultures and review antibiotics for appropriateness and modification accordingly. - Patients are notified of prescription changes. | ED pharmacist | • EHR review  • Telephone follow-up |
| Okere, 2015 [57] | Pharmacist-led MTM and medication reconciliation in collaboration with the ED | Evidence gap, Opportunity for pharmacists, Supporting evidence, Unique ED opportunities/ challenges, Cost, Policy | • Letter/ Email to communicate with primary care providers (a copy is also given to patients) • Patients' primary/ secondary pharmacy prescriptions • Patient’s medication and medical history profile | - Medication therapy management and reconciliation service from a pharmacist in collaboration with the ED physician. - Communication with PCP for modified therapy. | Clinical pharmacist | • Face-to-face patient/caregiver interviews  • Communication with health providers through telephone/Email |
| Briggs, 2015 [58] | Emergency department medication review (EDMR) by clinical pharmacist | Evidence gaps, Opportunity for pharmacists, Unique ED opportunities/ challenges, Cost | • The Identification of Seniors at Risk (ISAR) screening tool • Beers criteria list and Anticholinergic burden scale • Patient's medication list from their general practitioner • 6-12 months community pharmacy dispensing history • Fax | - CP conduct medication reconciliation and interviews the patients about their medication use (compliance, overuse, duplication, missing therapy, interactions, older adult dosing, adverse reactions, and precautions). | Experienced clinical pharmacist | • Face-to-face for patients  • Communication with health providers through letters |
| Falconieri, 2014 [59] | Transition of care program for DVT patients in the ED | Evidence gaps, Unique ED opportunities/ challenges, Cost, Policy, Equity and access to care | • General DVT and medication specific educational handouts • A documentation program  • A computerized physician order entry system linked with the medication administration record (MAR) • A patient satisfaction survey • Telephone | - Discharge anticoagulant is selected based on history, physical exam, labs, and insurance coverage. - Phone follow-up to assess efficacy, adherence, side effects, follow-up appointments. | Two ED clinical pharmacists, 2 anticoagulation clinical pharmacists, and a PGY2 pharmacy cardiology resident. Education was given to all providers | • Electronic orders  • Face-to-face patient education  • Telephone follow-up |
| Dumkow, 2014 [60] | Multidisciplinary CFU | Evidence gaps, Opportunity for pharmacists, Unique ED opportunities/ challenges | • Computerized decision support software to alert the pharmacists of culture results • Patient-specific report containing the patient's data  • Telephone  • Mail | - Positive cultures and discharge antimicrobial therapy were reviewed. - If a therapeutic change was determined necessary, the pharmacist created a patient-specific report with the recommended therapy to be discussed with the ED physician. | PGY1 pharmacy resident, with support and oversight from infectious diseases and ED pharmacy specialists | Telephone/ mail follow-up |
| Angoulvant, 2013 [61] | Parent therapeutic education on antibiotics | Evidence gaps, Opportunity for pharmacists, Unique ED opportunities/ challenges, Cost, Policy | • Interview grid for standardization • Different forms of oral solutions for practice demonstration • Illustrated information sheets • Drawing tools (Barrows cards) • Telephone • Questionnaire for phone follow-up | - Clinical pharmacists delivered education with parents on the preparation, administration, undesirable side effects and their management, and the adherence of antibiotics. | Five pharmacists trained in therapeutic education | Face-to-face educational session for each parent/child with phone follow-up |
| Cesarz, 2013 [62] | Emergency department discharge prescription interventions by emergency medicine pharmacists | Evidence gap, Opportunity for pharmacists, Supporting evidence, Unique ED opportunities/ challenges | • CPOE (Computerized Provider Order Entry) system | - EMP would review electronic prescriptions to prevent medication errors and optimize medication therapy upon ED discharge. | Four EM pharmacists. Education was provided to EM pharmacists on study procedures and documentation | Prescription review |
| Davis, 2012 [63] | A pharmacist-managed deep vein thrombosis (DVT) treatment program | Opportunity for pharmacists, Cost | • McKesson InterQual solution. • Warfarin brochure and international kit for bridging (educational literature, a sharps container, an alcohol swab, and an instructional video). | - Recommendations regarding appropriate anticoagulation to ED physicians by pharmacists. - Counseling, education, and follow-up appointments to the warfarin clinic for patients. | A postgraduate year 1 pharmacy practice resident, clinical pharmacist | • Education was delivered to the patient face-to-face  • The medications were either delivered by the pharmacist to the bedside or the patient was instructed on where to obtain the medications |
| Randolph, 2011 [64] | Pharmacist-managed ED culture review | Opportunity for pharmacists, Supporting evidence, Unique ED opportunities/ challenges, Policy | • Patients' medical records • Telephone  • Positive culture and susceptibility reports | - Positive cultures and empirically prescribed antimicrobial therapy were reviewed by pharmacists - If necessary, a new prescription is called in to the patient's outpatient pharmacy. | ED pharmacists | • EHR review |
| Mortimer Dip, 2010 [65] | Aged care pharmacist in the ED to assess elderly patients. | Evidence gap, Opportunity for pharmacists, Supporting evidence, Unique ED opportunities/ challenges, Cost | • Medication Action Plan (MAP) documentation system • Medication list | - The ACP performed medication reconciliation of the orders, identification of medication-related issues of patient, and education and referrals where warranted. | A specialist Aged Care Pharmacist (ACP) | • Medication review  • Face-to-face education |

***Abbreviations:*** *ADE: Adverse Drug Event; APP: Advanced Practice Provider; BPMH: Best Possible Medication History; CDTM: Collaborative Drug Therapy Management; CMR: Comprehensive Medication Review; CPA: Collaborative Practice Agreement; DRP: Drug-Related Problem; ED: Emergency Department; EHR: Electronic Health Record; EM: Emergency Medicine; EMP: Emergency Medicine Pharmacist; GEDI: Geriatric Emergency Department Intervention; GP: General Practitioner; ID: Infectious Diseases; MDT: Multidisciplinary Team; MO: Medical Officer; MRP: Medication-Related Problem; MTM: Medication Therapy Management; nPEP: Non-Occupational Post-Exposure Prophylaxis; PCP: Primary Care Provider; PGY: Postgraduate Year; PIM: Potentially Inappropriate Medication; PPDPP: Partnered Pharmacist Discharge Prescription Planning; PPMC: Partnered Pharmacist Medication Charting; RAIDS: Rapid Administration of Intravenous Antimicrobials by an Infectious Disease Specialist; SHPA: Society of Hospital Pharmacists of Australia; SMTP: Shared Medication Treatment Plan; SSU: Short Stay Unit; STOPP/START: Screening Tool of Older Persons' potentially inappropriate Prescriptions / Screening Tool to Alert doctors to the Right Treatment; TCM: Time-Critical Medicine; TIDieR: Template for Intervention Description and Replication; TOC: Transitions of Care; UTI: Urinary Tract Infection; VTE: Venous Thromboembolism; VOCA: Victims of Crime Act.*

***Table S7:*** *Showing the summary of the TIDieR items (7-12) for the pharmacy-supported transitional care interventions in the emergency department*

|  |  |  |  |  |  |  |
| --- | --- | --- | --- | --- | --- | --- |
| Author,  Publication year | **Item 7. Where** | **Item 8. When and how much** | **Item 9. Tailoring** | **Item 10. Modifications** | **Item 11. How well (planned)** | **Item 12: How well (actual)** |
| Kaucher, 2025 [2] | A 550-bed urban, acute care hospital and vertically integrated health-care system with an on-site STD (Sexually Transmitted Diseases) clinic and regional referral center for victims of sexual assault | • Quantification:   - Total interventions: 147 patients (100%) received the intervention   • Duration: Not reported | Not reported | Financial assistance was later obtained through the Victims of Crime Act (VOCA) after EM clinical pharmacists identified that patients may have an inability to obtain ongoing nPEP due to costs. | Not reported | 24% of the total patients attempted to be contacted were reached |
| Boot, 2024 [3] | Adult ED of a 566-bed acute care hospital (more than 56,000 annual visits) | • Quantification:   - Total interventions: 100 cultures (100%) were reviewed in the post-intervention group - Therapeutic interventions: 28 patients received therapeutic interventions (28.0%)   • Duration: 11-27 minutes, depending on whether it requires an intervention | Not reported | Not reported | Pharmacists were trained and certified as smoking cessation counsellors | Not reported |
| Reilly, 2024 [4] | Tertiary referral hospital ED in regional Queensland, Australia. | • Quantification:   - Total interventions: pharmacists reviewed 1214 older adults (100%) - Therapeutic interventions: 447 older adults (36.8%) had STOPP recommendations   • Duration: Not reported | Not reported | No reported | Not reported | 83% of participants with STOPP criteria recommendations had some of these medications ceased, and 69% had any START criteria recommended medication commenced |
| Lee, 2024 [5] | ED short stay unit at a tertiary hospital (19,000 annual visits) | • Quantification:   - Total interventions: A total of 147 discharge prescriptions (100%) were collaboratively developed   • Duration: Not reported | Not reported | Not reported | A medical officer was responsible for the final verification of the discharge plan and prescription printing | Not reported |
| Selman, 2024 [6] | Academic tertiary care center | • Quantification:   - Total interventions: 577 patients (100%) were reviewed by the ED pharmacists - Therapeutic interventions: 309 patients (53.7%) received a pharmacist recommendation   • Duration: Not reported | Not reported | Not reported | Not reported | Not reported |
| Martínez, 2024 [7] | Emergency department (37,000 annual visits) | • Quantification:   - Total interventions: 500 patients (100%) were reviewed by the clinical pharmacists - Therapeutic interventions: 79 interventions were recorded   • Duration: Not reported | Not reported | Not reported | An independent and blinded pharmacist conducted patient interviews using a structured form | 82% of pharmacist recommendations were accepted |
| Maleki, 2024 [8] | Three EDs within the healthcare network, with comprehensive 7-day extended-hours ED clinical pharmacy service | • Quantification:   - Total interventions: 105 high-needs patients (100%) were reviewed by ED pharmacists and received a Best Possible Medication History (BPMH) prior to hospital admission - Therapeutic interventions: 74 patients (70.5%) had at least one error identified   • Duration: Not reported | Not reported | Not reported | • Prescribing errors were extracted from those who were not involved in the patients’ care  • A 10% random sample was assessed by a blinded ED physician. Both assessors were blinded to patient allocation | Not reported |
| Sofeso, 2024 [9] | Urban academic medical center (more than 130,000 annual visits) | • Quantification:   - Total interventions: 534 discharge prescriptions (100%) were reviewed by the pharmacists - Therapeutic interventions: 183 of the prescriptions (34.3%) were intervened on by the pharmacists   • Duration: 81% of the reviews took <15 minutes | Not reported | No reported | Not reported | Pharmacists intervened on 183 discharge prescription antibiotics reviewed (34.3%) |
| Tran-Nguyen, 2024 [10] | Not reported | • Quantification:   - Total interventions: 120 patients (100%) received the medication review and discharge support - Therapeutic interventions: 107 interventions were documented   • Duration: Not reported | Not reported | Not reported | Not reported | Not reported |
| Atey, 2024 [11] | A 490-bed teaching and referral public hospital. | • Quantification:   - Total interventions: A total of 230 patients (100%) in the PPMC group received pharmacist-supported BPMH   • Duration: Not reported | Not reported | Not reported | Pharmacists were trained to deliver the intervention | 97% of VTE risk assessments were completed in the ED |
| Wang, 2024 [12] | A large academic medical center with an ED consisting of a total of 30 beds (40,000 annual visits) | • Quantification:   - Total interventions: 26 patients (100%) received the pharmacist-supported follow-up   • Duration: Pharmacy residents reported spending around 30 minutes/ day on the patient assessment and outreach | Not reported | A revision was added to the protocol, which states that if a pharmacy resident is unavailable, a clinical ED pharmacist implements the protocol by adding the deprescribing procedure to the original culture callback process | Not reported | Not reported |
| Kofoed, 2023 [13] | Regional network of five predominantly rural EDs, staffed by approximately 50 clinicians (70,000 annual visits) | • Quantification:   - Total interventions: 30 patients (100%) were reviewed   • Duration: Not reported | Not reported | No reported | Clearly defined procedures for reviewing patients and facilitating communication were established, including example scripts for patient interactions. | Not reported |
| Pham, 2023 [14] | An ED in a medical center (over 50,000 annual visits) | • Quantification:   - Total interventions: 136 urine cultures (100%) were reviewed - Therapeutic interventions: 20 (14.7%) cultures required interventions   • Duration: Not reported | Not reported | Not reported | Not reported | Not reported |
| Jovevski, 2023 [15] | Veterans Affairs (VA) ED, a Level Ia highest complexity VA facility, urban, tertiary care facility with academic affiliations (approximately 26,625 annual visit) | • Quantification:   - Total interventions: 149 patients (100%) received pharmacist-supported medication reconciliation - Therapeutic interventions: 35 (23.5%) had at least one PIM identified   • Duration: Not reported | Not reported | Not reported | Not reported | Not reported |
| Andrade, 2023 [16] | Single-center, a 464-bed urban community teaching hospital with a 42-bed adult ED (65,000 annual visits) | • Quantification:   - Total interventions: 70 patients (100%) received pharmacist-supported follow-up for their cultures - Therapeutic interventions: 28 (35.9%) out of 78 culture results supported to pharmacist interventions   • Duration: Not reported | Not reported | Not reported | Competency was evaluated through daily review and feedback from an Emergency Medicine Pharmacotherapy Specialist on EMR progress notes completed by pharmacy residents. | Not reported |
| Dunn, 2023 [17] | An ED in a medically underserved area. No further information on setting were reported | 28 interventions were done  • Quantification:   - Total interventions: 70 patients (100%) received pharmacist-supported follow-up for their cultures - Therapeutic interventions: 28 (35.9%) out of 78 culture results supported to pharmacist interventions   • Duration: Not reported | Not reported | Not reported | Not reported | 58% of patients in the intervention group were reached |
| Atey, 2023 [18,19,20] | 490-bed tertiary, teaching and  referral public hospital (over 63,000 annual visits) | • Quantification:   - Total interventions: 309 patients (100%) received the intervention   • Duration: The estimated time to deliver PPMC for a single patient was 75 minutes | Not reported | Not reported | Pharmacists were trained to deliver the intervention | Not reported |
| Zhao, 2023 [21] | A single health system that includes six hospitals | • Quantification:   - Total interventions: 72 patients (100%) had urine cultures reviewed   • Duration: Not reported | Not reported | Not reported | Not reported | Not reported |
| Benson, 2023 [22] | 12-bed, rural hospital ED. | • Quantification:   - Total interventions: 105 urine cultures (100%) were reviewed by pharmacists, with 65 of them belonging to patients who had antibiotics prescribed - Therapeutic interventions: 26 of 65 patients (40.0%) who had antibiotics in the post-intervention period had their antibiotics discontinued or changed.   • Duration: Not reported | Not reported | Not reported | Not reported | Not reported |
| Ng, 2022 [23] | ED short stay and smoking cessation clinic. This acute care hospital belongs to a regional health care cluster with an emergency medicine academic clinical program (150,000 annual visits) | • Quantification:   - Total interventions: 47 patients (100%) received the smoking cessation service   • Duration: The estimated time for the bedside counseling session was up to 45 minutes per patient. For the outpatient follow-up session, it was up to 20 minutes three times weekly. | Not reported | Not reported | Pharmacists were trained and certified as smoking cessation counsellors | Not reported |
| Nymoen, 2022 [24] | Norwegian ED, a local, urban hospital covered by a 0.5 full-time  equivalent pharmacist position | • Quantification:   - Total interventions: 405 (100%) patients received the allocated intervention - Therapeutic interventions: 646 drug-related problems were identified   • Duration: Not reported | Not reported | An exclusion criterion was originally added regarding terminally ill patients with short life expectancy. However, it was later removed. | A checklist and a validated medication review tool were used to standardize medication reconciliation | Not reported |
| Ogilvie, 2022 [25] | Emergency department of a medium-sized (170-bed) metropolitan hospital in Australia. ED had two full-time equivalent (FTE) pharmacists working from 7.30 am to 5 pm Monday to Friday. | • Quantification:   - Total interventions: 35 patients (100%) received collaborative pharmacist prescribing - Therapeutic interventions: 412 orders were written for the 35 patients   • Duration: Not reported | Not reported | Not reported | Medication charts were audited retrospectively by an independent auditor, using validated audit forms. | Not reported |
| Stewart, 2021 [26] | A community academic hospital located in Northwest Detroit. | • Quantification:   - Total interventions: 116 patients were screened, with 44 (37.9%) completing at least one clinic visit   • Duration: Not reported | Each patient received individualized care with an emphasis on overcoming medication access issues and other medication-related barriers | Not reported | Not reported | 44 patients (37.9%) presented to at least one TCC visit |
| Lineberry, 2021 [27] | An urban academic medical center with approximately (80,000 annual visits) | • Quantification:   - Total interventions: 378 prescriptions (100%) were reviewed - Therapeutic interventions: 70 prescriptions (18.5%) required an intervention   • Duration: median of 12 minutes | Not reported | Not reported | Not reported | Four prescriptions (2.5%) were considered to have incomplete interventions after discharge, due to the inability to contact the patient |
| Celikkayalar, 2021 [28] | ED ward, an acute, short-term care unit with 16 beds, part of an ED providing primary and specialized secondary health care (24,000 annual visits) | • Quantification:   - Total interventions: 855 patients (100%) were reviewed - Therapeutic interventions: 113 inappropriate prescribing (IP) events were identified in 83 patients (9.7%)   • Duration: Not reported | Not reported | Not reported | Pharmacists were specially trained to deliver the intervention | Not reported |
| Castillo, 2021 [29] | Emergency department of a multispecialty academic medical center (65,000 annual visits). | • Quantification:   - Total interventions: 1648 patients (100%) had their discharge prescriptions reviewed - Therapeutic interventions: 120 patients (7.3%) received interventions   • Duration: Not reported | Not reported | Not reported | Not reported | Not reported |
| Rainess,2021 [30] | A large community hospital with a high-volume ED (100,000 annual visits) | • Quantification:   - Total interventions: 91 patients (100%) received the review   • Duration: Not reported | Not reported | Not reported | Not reported | Not reported |
| Olson, 2020 [31] | An adult urban level 1 trauma academic medical center (72,000 annual visits) | • Quantification:   - Total interventions: 139 patients (100%) had their cultures reviewed - Therapeutic interventions: 139 patients (100%) had documented recommendations   • Duration: Not reported | Not reported | Not reported | Not reported | Not reported |
| Roels, 2020 [32] | A 297-bed community hospital that serves several counties in south-central Indiana. (48,550 annual visits) | • Quantification:   - Total interventions: CPA was used in 1094 cultures (100%) - Therapeutic interventions: 394 cases (36.0%) required intervention by pharmacists   • Duration: Average time per case 21.8 minutes (calculated from the average time per day and the average number of cases per day) | Not reported |  | Not reported | The CPA was used in 1094 of 1589 eligible cultures (68.8%) |
| Lim, 2020 [33] | A hospital in Australia. No further information reported | • Quantification:   - Total interventions: 120 patients (100%) received the rivaroxaban pack and pharmacist follow-up   • Duration: Not reported | Not reported | Not reported | Not reported | All 120 patients (100%) received pharmacist follow-up via telephone for education, with referral to the clinic |
| Tweedle, 2020 [34] | Tertiary pediatric ED (160,000 annual visits). Each ED is staffed 24 hours a day by pediatric emergency physicians. | • Quantification:   - Total interventions: A total of 1863 cultures were reviewed by pharmacists. Out of those, 852 cultures (100%) were analyzed. - Therapeutic interventions: 852 positive cultures (100%) required interventions   • Duration: Not reported | Not reported | Not reported | Not reported | Not reported |
| Wu, 2020 [35] | 10 hospital-based EDs and 3 freestanding EDs in a large, integrated health system (16,000 to 93,000 annual visits) | • Quantification:   - Total interventions: A total of 7664 ED encounters (100%) were reviewed - Therapeutic interventions: 3049 clinical interventions were made across ED encounters   • Duration: Not reported | Not reported | Not reported | • Therapeutic guides were created to ensure standardized processes across all sites.  • A process map was utilized to determine next steps for therapy intervention, patient counseling, and EHR documentation | Not reported |
| Houlind, 2020 [36] | The hospital has approximately 14,000 medical admissions each year, of which 85% are acute admissions. The ED has a 29-bed medical ward handling all acute admissions, and a separate emergency room (ER) handling all minor injuries and traumas. Patients are referred to the ED by general practitioners (GPs), medical helpline, or emergency call. | • Quantification:   - Total interventions: The intervention was started in 60 patients and completed in 50 (83%). - Therapeutic interventions: 131 prescription changes were recommended by the clinical pharmacist   • Duration: Not reported | Not reported | Not reported | Not reported | Out of sixty patients included from the ED, the intervention was completed before discharge for 50 patients (83.0%) |
| Layman, 2020 [37] | A medical center providing a full range of patient care services to approximately 196,000 veterans. The center includes a 250 inpatient facility, primary care clinics, and a variety of specialty clinics. | • Quantification:   - Total interventions: 114 patients (100%) were included in the analysis in the 2016 TCC arm, while 32 were included in the propensity-matched cohort - Therapeutic interventions: 372 interventions were made in the retrospective TCC arm, whereas 198 were made in the propensity-matched TCC cohort   • Duration: 30 minutes/ appointment | • Educational tools and consults were provided based on the individual patient's needs • TCC services were expanded to include telehealth appointments to increase access for rural and/or homebound patients | Due to its success, the clinic was expanded to a thrice-weekly clinic with 4 new transitional care clinical pharmacy specialists TCC services were expanded to include telehealth appointments to increase access for rural and/or homebound patients | Not reported | Not reported |
| Pearson, 2020 [38] | The Seniors Clinic comprises two multi-disciplinary primary care sites for older people in the Denver-Metro area and serves around 3,500 patients with 13 medical providers and three embedded clinical pharmacists | • Quantification:   - Total interventions: 122 out of 143 patients (85.4%) received the full telephonic pharmacist intervention - Therapeutic interventions: Medication discrepancies were identified in 105 patients (86.0%)   • Duration: Average time spent on encounter was 52 minutes | Not reported | Not reported | Not reported | 21 of the 170 high-risk patients (12%) were unable to be reached for medication reconciliation. Pharmacists successfully completed medication reconciliation with 122 of these patients(71.8%) |
| Kitchen, 2020 [39] | 3 British Columbian emergency departments, including 1 tertiary care referral center and 2 urban community hospitals. (185,000 combined annual visits) | • Quantification:   - Total interventions: 6403 patients (100%) received the medication review   • Duration: Not reported | Not reported | Not reported | Not reported | Not reported |
| Shealy, 2020 [40] | A tertiary teaching hospital (95,000 annual visits) | • Quantification:   - Total interventions: 63 discharged ED patients (100%) had pharmacist-supported review - Therapeutic interventions: Medications were initiated or modified in 33 patients (52.4%)   • Duration: Not reported | Not reported | Not reported | Not reported | 12 patients (19%) in the post-implementation period were not reached |
| Loborec, 2020 [41] | Urban academic medical center (72,000 annual visits) | • Quantification:   - Total interventions: 86 positive microbiologic test results (100%) were reviewed by the privileged pharmacists - Therapeutic interventions: Interventions were made in 63 patients (73.3%)   • Duration: Not reported | Not reported | Not reported | • A board-certified infectious diseases (ID) physician reviewed each microbiologic test result to determine the appropriateness of ED-SPP interventions.  • Training was a prerequisite to the intervention. | 2 out of 86 interventions (2.3%) were determined to be unnecessary |
| Eswaran, 2020 [42] | Urban academic hospital-based ED (91,000 annual visits). The emergency department is staffed 24 hours a day, 7 days a week, 365 days a year by a dedicated clinical pharmacist and social worker | • Quantification:   - Total interventions: 669 unique ED visits (100%) occurred within the ED - Therapeutic interventions: 168 THN kits (25.1%) were dispensed   • Duration: Education and dispensing of the kit took an average of 15 minutes per patient | Not reported | Not reported | A manual log of dispensed naloxone was maintained to verify the accuracy of electronic recordkeeping. | Not reported |
| Giruzzi, 2020 [43] | Community hospital ED | • Quantification:   - Total interventions: 392 patients (100%) had their positive cultures reviewed   • Duration: Not reported | Not reported | Not reported | Not reported | Positive cultures were evaluated on a daily basis in approximately 98% of the time during the study period. |
| Cabilan, 2019 [44] | The ED is in a public, metropolitan hospital (61,000 annual visits) | • Quantification:   - Total interventions: 14 patients (100%) received complete discharge medication counseling, which included verbal and written information, bedside discussion, and the teach-back method.   • Duration: Not reported | Not reported | Not reported | Not reported | Not reported |
| Schwab, 2019 [45] | A community-based Level II Trauma hospital serving patients, primarily in a medically underserved community | • Quantification:   - Total interventions: 131 patients (100%) were assessed and/ or managed by the multidisciplinary team, including an ED pharmacist   • Duration: Not reported | Selection of the OAC was determined primarily by the patient's insurance coverage, and exclusion based on any contraindication or warning such as renal dysfunction or drug-drug interaction. | The ED pharmacist expanded the protocol to include conversion from warfarin to OACs in patients with subtherapeutic International Normalized Ratios (INRs) | Not reported | Not reported |
| Fay, 2019 [46] | Two free-standing urgent care sites. Both centers offer comprehensive care for adults and children 12 hours per day, 7 days a week. (32,000 combined annual visits) | • Quantification:   - Total interventions: 150 patients (100%) discharged from urgent care were monitored under the pharmacist-supported stewardship protocol - Therapeutic interventions: 16 patients (10.7%) required a follow-up phone call by the pharmacist   • Duration: The median pharmacist time per intervention was 15 minutes | Not reported | Not reported | Not reported | Five out of 150 patients (3.33%) in the post-ASP period were lost to follow-up after 3 contact attempts |
| Southerland, 2011 [47] | An urban community ED with 30 beds. (55,000 annual visits) Recruitment later expanded to an affiliated academic tertiary care ED.  The study pharmacy was an independent community pharmacy with a contemporary practice model (combining traditional dispensing and compounding with personalized patient care services such as immunization, medication therapy management services, and point-of-care testing)  The study home health care agency was a regional agency selected due to its pre-existing relationship with the ED | • Quantification:   - Total intervention: 3 patients (100%) received the pharmacist-supported intervention - Therapeutic interventions: 3 MRPs were identified   • Duration: Not reported | Not reported | Due to low recruitment in the urban community emergency department (ED), several modifications were implemented. Recruitment was expanded to include an affiliated academic tertiary ED. In addition, the age inclusion criteria were lowered from 65 to 50 years. To further address enrollment barriers, a third service was introduced, offering either the pharmacy-only service or the combination of pharmacy and home health care service, excluding medication delivery. | Not reported | Only one patient out of the three enrolled patients (33.3%) was able to be reached for the Days 3 and 10 follow-ups |
| Lacy, 2018 [48] | The ED of a Midwestern health system | • Quantification:   - Total intervention: 308 out of 440 patients (70.0%) discharged from the ED were successfully contacted within 7 days - Therapeutic interventions: 153 of the calls (49.7%) resulted in at least one intervention   • Duration: Not reported | Not reported | Not reported | • A standardized script was developed and utilized by all callers to ensure consistency between calls.  • An electronic template for post-ED discharge follow-up calls was developed for EMR documentation | Medication reconciliation was completed during 87% of the phone calls |
| Santolaya-Perrín, 2018 [49, 56] | Emergency departments at four  Spanish hospitals | • Quantification:   - Total intervention: 323 patients (100%) were randomized to the intervention group - Therapeutic interventions: 581 PIPs and 317 DRPs were communicated with GPs   • Duration: Not reported | Not reported | Not reported | Not reported | The acceptance of recommendations varied by site (27% in site 1, 31% in site 2, 52% in site 3, and 53% in site 4) |
| Chu, 2017 [50] | An urban, community hospital (100,000 annual visits). Of the patients discharged from the ED, over half are either uninsured or underfunded | • Quantification:   - Total intervention: 41 patients (100%) received the Starter Pack, counselling, and follow-up   • Duration: Not reported | Not reported | Not reported | Not reported | Not reported |
| DiRenzo, 2017 [51] | The clot clinic is located within an academic, safety-net, Level I trauma center in a metropolitan city. The clinic takes place once a month for 4 hours and provides services for up to 12 outpatients with low-risk VTE treated with rivaroxaban. | • Quantification:   - Total intervention: 17 patients (100%) were seen in the pharmacist-managed clinic   • Duration: Not reported | Not reported | Pharmacists have added apixaban to their CPA to broaden the list of anticoagulants that can be prescribed. | A standardized question list and note template were utilized by pharmacists | Not reported |
| Hohl, 2017 [52] | One tertiary care and two urban community hospitals | • Quantification:   - Total intervention: 6416 patients (100%) were reviewed by pharmacists - Therapeutic interventions: 2284 patients (35.6%) were identified as having at least one adverse drug event   • Duration: Not reported | Not reported | Not reported | • Study operation procedures were developed and shared with participants and personnel  • An interdisciplinary team consisting of two chief physicians and three experienced clinical pharmacists classified all identified drug-related problems according to clinical relevance | Not reported |
| Zdyb, 2017 [53] | A 900-bed, tertiary care, academic medical center. The ED is a 55-bed level 1 trauma center (over 85,000 annual visits) | • Quantification:   - Total intervention: 151 patients (100%) received the pharmacist intervention - Therapeutic interventions: 14 of 108 patients (13.0%) who were reached by phone follow-up required an intervention   • Duration: Not reported | Not reported | Not reported | A standardized electronic form was used by the pharmacist (bedside documentation, callback documentation). | There was a 25.7% loss to follow-up rate |
| Hohner, 2016 [54] | A tertiary academic medical center is a Level I trauma center (70,000 annual visits) | • Quantification:   - Total intervention: 18 patients (100%) received the intervention - Therapeutic interventions: 9 patients (50.0%) had therapy additions recommended   • Duration: The mean time spent by pharmacists per patient was 28 minutes | Not reported | Not reported | A standardized form specific to the underlying disease was used to consistently address potential medication-related issues | Of the 18 patients who were referred to follow-up care, 5 successfully followed up with a pharmacist after ED discharge (27.8%) |
| Lingenfelter, 2016 [55] | A tertiary referral center (40,000 annual visits) | • Quantification:   - Total intervention: 180 patients (100%) with positive urine cultures were reviewed - Therapeutic interventions: 42 discharge prescriptions (23.0%) required interventions   • Duration: Not reported | Not reported | Not reported | Not reported | ED pharmacists managed to contact 35 of the 42 patients with identified inappropriate prescribing (83%). The rest were unable to be contacted |
| Okere, 2015 [57] | 380-bed community hospital Level II Trauma Center with (33,000 annual visits), with data also collected from the affiliated clinic system | • Quantification:   - Total intervention: 278 patients (100%) received the MRS service   • Duration: Not reported | Not reported | Not reported | Pharmacy technicians and students were trained to collect medication histories | Not reported |
| Briggs, 2015 [58] | A tertiary referral ED in New South Wales, Australia (70,000 annual visits) | • Quantification:   - Total intervention: 525 patients (100%) received the ED medication review   • Duration: Not reported | Not reported | Not reported | Not reported | 49% of the pharmacist's recommendations were accepted |
| Falconieri, 2014 [59] | Not reported | • Quantification:   - Total intervention: 15 patients (100%) received the intervention   • Duration: Not reported | Not reported | The FAST program’s documents were reviewed quarterly by pharmacists, with updates made as needed. One of those updates was pharmacists updating the inclusion criteria to be more detailed | • A structured evaluation and follow-up process was developed to support clinician decision-making  • Researchers assessed provider adherence to obtaining the recommended labs in the ED, as well as evaluating each component of the admission criteria  • Pharmacists were also given education before delivering the intervention | Only 2 (29%) of the patients discharged from the ED post-FAST were appropriate for discharge according to the admission criteria. |
| Dumkow, 2014 [60] | 802-bed teaching hospital with an existing ASP presence in inpatient and ED services. | • Quantification:   - Total intervention: 197 patients (100%) received the culture follow-up - Therapeutic interventions: 50 patients (25.5%) required therapeutic modification   • Duration: Not reported | Not reported | Not reported | Not reported | Not reported |
| Angoulvant, 2013 [61] | A tertiary pediatric hospital | • Quantification:   - Total intervention: 148 children and their parents (97.7%) attended the educational session   • Duration: Each session lasted 30 minutes | Not reported | Not reported | • The pharmacists delivering the education used an interview grid to ensure standardization of the sessions.  • Pharmacists were trained in therapeutic education | Patient data at day 14 were unable to be obtained for 21 (14%) patients in the intervention group |
| Cesarz, 2013 [62] | A 32-bed academic, tertiary care ED (45,000 annual visits). The ED is a level I burn and trauma center and the primary teaching site of an Emergency Medicine residency training program. | • Quantification:   - Total intervention: 674 discharge prescriptions (100%) were reviewed - Therapeutic interventions: 68 prescriptions (10.1%) were intervened upon   • Duration: 83.8% of interventions took <5 minutes | Not reported | Not reported | • Standardized forms were developed and  • Education was provided to EM pharmacists on study procedures and documentation, including guidance on how to discriminate between medication error and optimization of therapy | 53.4% of all prescriptions were captured in this 3-week study period (when the pharmacists were on duty). |
| Davis, 2012 [63] | A 1500 + bed tertiary care, academic, medical center | • Quantification:   - Total intervention: 14 ED-discharged patients (100%) received the MTM service   • Duration: Not reported | In case the patient could not pay for these essential anticoagulants, the pharmacists partnered with a group of hospital administrators and physicians who reviewed each case to determine whether a waiver of the cost of the medications or copay could be allowed. | Services were expanded to inpatients after identifying that the inpatient setting could benefit from the intervention. | • The pharmacists gave the participants a standard educational kit and brochure on anti-coagulation  • A predetermined institution-specific protocol that directed warfarin dosing was utilized | Following hospital discharge or release from the ED, 91% of patients attended their outpatient follow-up appointment at the warfarin clinic. |
| Randolph, 2011 [64] | Not reported | • Quantification:   - Total intervention: 2361 cultures (100%) were reviewed by pharmacists - Therapeutic interventions: 355 cases (15.0%) required modifications   • Duration: Average time per review was 15 minutes | Not reported | Not reported | Not reported | Not reported |
| Mortimer Dip, 2010 [65] | Patients presenting to DEM   (35,967 annual visits) | • Quantification:   - Total intervention: 101 elderly patients (100%) received the intervention - Therapeutic interventions: 81 MRPs were identified in 51 (70.0%) out of 73 admitted patients, and 24 MRPs were found in 18 patients (64.0%) out of 28 discharged patients   • Duration: Not reported | Not reported | Not reported | Not reported | Not reported |

***Abbreviations:*** *ADE: Adverse Drug Event; APP: Advanced Practice Provider; BPMH: Best Possible Medication History; CDTM: Collaborative Drug Therapy Management; CMR: Comprehensive Medication Review; CPA: Collaborative Practice Agreement; DRP: Drug-Related Problem; ED: Emergency Department; EHR: Electronic Health Record; EM: Emergency Medicine; EMP: Emergency Medicine Pharmacist; GEDI: Geriatric Emergency Department Intervention; GP: General Practitioner; ID: Infectious Diseases; MDT: Multidisciplinary Team; MO: Medical Officer; MRP: Medication-Related Problem; MTM: Medication Therapy Management; nPEP: Non-Occupational Post-Exposure Prophylaxis; PCP: Primary Care Provider; PGY: Postgraduate Year; PIM: Potentially Inappropriate Medication; PPDPP: Partnered Pharmacist Discharge Prescription Planning; PPMC: Partnered Pharmacist Medication Charting; RAIDS: Rapid Administration of Intravenous Antimicrobials by an Infectious Disease Specialist; SHPA: Society of Hospital Pharmacists of Australia; SMTP: Shared Medication Treatment Plan; SSU: Short Stay Unit; STOPP/START: Screening Tool of Older Persons' potentially inappropriate Prescriptions / Screening Tool to Alert doctors to the Right Treatment; TCM: Time-Critical Medicine; TIDieR: Template for Intervention Description and Replication; TOC: Transitions of Care; UTI: Urinary Tract Infection; VTE: Venous Thromboembolism; VOCA: Victims of Crime Act.*

***Table S8:*** *Showing the secondary outcome descriptions of the included studies*

| **First author,**  **Publication year** | **Intervention brief name** | **Secondary outcome description** |
| --- | --- | --- |
| **Kaucher, 2025 [2]** | ED pharmacist-led nPEP counseling for sexual assault survivors | • HIV seroconversion occurrence  • nPEP (nonoccupational postexposure prophylaxis) medication adherence  • Incidence of nPEP related ADE (adverse drug events) |
| **Boot, 2024 [3]** | Pharmacy-led ED culture callback program | • Time from culture results to review by nursing or pharmacy  • Return visits to the ED and admissions within 30 days due to infection   • Total time pharmacist spent reviewing cultures |
| **Lee, 2024 [5]** | Partnered pharmacist discharge prescription planning (PPDPP) | • The difference in the time taken from discharge decision to prescription given to patients and to actual discharge  • The rate of high-risk medicines prescribed.  • Availability of BPMH at the time of discharge prescription review by pharmacists. |
| **Selman, 2024 [6]** | ED pharmacist led high-risk drugs' deprescribing for older adults | • Timing of follow-up PCP visit |
| **Martínez, 2024 [7]** | Clinical pharmacists' integration into the ED discharge process | • Re-consultations to any center within 30 days after discharge   • Patients satisfaction |
| **Maleki, 2024 [8]** | Pharmacist-led review of high needs patients in the ED | • Number of patients with at least one medication error  • Impact of ED clinical pharmacy service on time to BPMH   • Time to pharmacist medication reconciliation |
| **Sofeso, 2024 [9]** | Prospective pharmacist review of discharge antibiotics for urinary tract infections in the ED | • Individual components of the composite endpoint  • Percentage of orders a pharmacist was consulted on prior to order placement  • Number of total orders reviewed by a pharmacist  • Percent of printed prescriptions which were not reviewed by an EM pharmacist  *Balancing metric*:    • Time spent by pharmacist per order reviewed |
| **Tran-Nguyen, 2024 [10]** | Preventing readmissions in older adults: a pharmacist-led ED collaborative | • Types of intervention |
| **Wang, 2024 [12]** | A pharmacist-driven deprescribing protocol for negative urine and sexually transmitted infection cultures | • Urinalysis characteristics of study groups  • Patient receipt of oral antibiotics on discharge, by study group |
| **Kofoed, 2023 [13]** | Pharmacist-driven follow-up results  (FUR) process | • Return visits to the ED with diagnosis of urinary symptoms  or serious infection within 30 days of the original visit |
| **Pham, 2023 [14]** | Pharmacist-led, urinary culture follow-up after discharge from the emergency department | • Rate of documentation of intervention  • Rate of appropriate interventions made (antibiotic choice, dosing, and duration)  • Repeat ED visits within 30 days |
| **Jovevski, 2023 [15]** | Ed pharmacist-led med reconciliation & deprescribing for high-risk seniors | • 30-day primary care follow-up  • 7 and 30-day hospitalizations  • 60-day mortality |
| **Atey, 2023 [18]** | Partnered pharmacist medication  charting (PPMC) | • Use of at least one PIM on hospital discharge   • Number of PIMs in each group |
| **Zhao, 2023 [21]** | Emergency department clinical pharmacist integration within a discharge urine culture follow-up program | • Unplanned admission or ED revisit within 14 and 30 days with a chief complaint related to the initial ED visit  • All-cause hospital admission or ED revisit within 14 and 30 days of index encounter  • Acceptance rate of pharmacist recommendations  *Post-hoc analyses*:  • UTI management appropriateness upon ED discharge |
| **Ng, 2022 [23]** | ED SSU smoking cessation service | • Reduction in the number of cigarettes smoked |
| **Nymoen, 2022  [24]** | Systematic medication review in emergency department | • Proportion of patients with an unplanned contact with hospital within 180 days after inclusion stay discharge  • Number of unplanned contacts with hospital per patient within 12 months after inclusion  stay discharge  • Time to next unplanned contact with a hospital within 12 months after inclusion stay  discharge |
| **Lineberry, 2021 [27]** | Pharmacist-targeted discharge prescription review in an emergency department | • Type and frequency of MRPs identified |
| **Castillo, 2021 [29]** | Discharge prescription optimization by emergency medicine pharmacists | • Correlation between high-risk criteria and EM pharmacist intervention rate   • Health-system ED and/or hospital readmission rates within 30 days from index ED visit between patients with and without EM pharmacist review. |
| **Rainess, 2021 [30]** | Addition of a clinical pharmacist service to a midlevel provider-driven culture follow-up program | • Optimal antibiotic choice, dose, and duration  • Return to the ED within 30 days due to infection |
| **Olson, 2020 [31]** | Pharmacist-initiated culture review process | • Incidence of treatment failure within 30 days of ED visits  • Incidence of hospital admission within 30 days of ED visits  • Percentage of all accepted pharmacist recommendations |
| **Roels, 2020 [32]** | Collaborative Practice Agreement (CPA) | • Average number of culture cases reviewed per day  • Average time spent daily reviewing culture data  • Number of cases not requiring further action under the CPA because treatment is appropriate  • Type of intervention used under the CPA  • Number of culture cases unable to be executed under the CPA, reason for CPA exclusion  • ED provider satisfaction with the CPA |
| **Tweedle, 2020 [34]** | Review of positive cultures by clinical pharmacists | • Time from positive culture result to time to initial review of  positive culture result  • Type and rate of interventions made in both the nurse-driven and pharmacist-driven periods |
| **Houlind, 2020 [36]** | A collaborative medication review and deprescribing intervention in an ED | • Change in the Assessment of Underutilization Index score from hospital admission to 30 days after discharge   • The percentage of patients where it was possible to complete the intervention before discharge without affecting hospitalization time  • The agreement between the pharmacist’s suggestions and the geriatrician’s intervention |
| **Pearson, 2020 [38]** | Post ED discharge telephonic outreach and assessment by a clinical pharmacist, with triaging to other staff if necessary | • The proportion of patients with at least one repeat ED visit, hospitalization, or death within 90 days of ED discharge   • Rates of UCHealth Seniors Clinic follow-up within 30 days of ED discharge  • Rates of medication discrepancies among the intervention cohort |
| **Kitchen, 2020 [39]** | Medication review delivered by pharmacists to ED patients | • Differences in the total general practitioner visits per 1000 patients per month  • Differences in the total ED visits per 1000 patients per month   • Patient outcomes per person-months 12 months before vs 12 months after the intervention   • Patient outcomes per person-week 52 weeks before vs 52 weeks after the intervention |
| **Shealy, 2020 [40]** | Pharmacist-Driven Culture and STI Testing Follow-Up Program in the Emergency Department | • Time from ED discharge to first contact attempts  • Proportion of patients with a repeat ED encounter within 30 days of the index encounter  • Proportion of patients with 30-day hospital readmission  • Antimicrobial prescribed during outpatient follow-up. |
| **Giruzzi, 2020 [43]** | Antimicrobial Stewardship pharmacist culture review service | • Median time from culture finalization to review  • Number of appropriate empiric antimicrobial selections,  duration, dose, and frequency per current IDSA guideline recommendations  • Number of appropriate empiric antimicrobial dosing based on renal function  • 30-day ED return visit or hospital admission rates after index visit |
| **Cabilan, 2019 [44]** | Pharmacist-led discharge medication counselling | • ED re-presentation within 48 hours  • Length of stay in SSU |
| **Schwab, 2019 [45]** | Multidisciplinary anticoagulation initiative in the ED | • All-cause 30-day re-admission   • INR value ranges for patients on admission warfarin   •  ADE at 2, 14, and 30 days |
| **Fay, 2019 [46]** | Pharmacist-led antimicrobial stewardship program (ASP) in urgent care. | • Number of patients who required a follow-up call  • Time to follow-up contact  • Urgent care or ED revisit within 72 hours  • Hospital admission within 30 days |
| **Southerland, 2011 [47]** | Inter-professional transitions of care services for older adults discharged from the ED | • Adherence assessment  • Number of ED revisits  • Number of hospital admission at day 30 |
| **Lacy, 2018 [48]** | A pharmacy-driven post-emergency department (ED) transitions of care program | • Rates of repeat ED visits within 30 days  • Rates of urgent care visits within 30 days  • Rates of hospital admissions within 30 days  • Rates of follow-up clinic visits within 30 days |
| **Chu, 2017 [50]** | A rivaroxaban program for acute venous thromboembolism upon emergency department discharge | • 90-day readmission rates due to bleeding or adverse events |
| **DiRenzo, 2017 [51]** | Outpatient treatment of VTE with rivaroxaban in the pharmacist-managed clinic | • Individual components of the primary outcome  • Number of hospitalizations after VTE diagnosis  • Adverse events  • Morisky medication adherence score |
| **Hohl, 2017 [52]** | Clinical pharmacist led systematic medication review | • ED revisits within seven days   • Hospital admission   • Odds of exceeding expected length of stay in patients aged 60-79   • Odds of exceeding expected length of stay in other age groups  • Mortality |
| **Zdyb, 2017 [53]** | Anticoagulation discharge education provided by ED pharmacists | • Number of patients with documented healthcare utilization within 90 days for a reason related to their anticoagulation therapy   • Percentage of patients who scheduled appropriate follow-up   • Rates of bleeding and clotting |
| **Briggs, 2015 [58]** | Emergency department medication review (EDMR) by clinical pharmacist | • Rate of readmission  • Length of stay and admission to an aged care facility at 4 months  • Rate of general practitioner acceptance of pharmacist recommendations |
| **Falconieri, 2014 [59]** | Transition of care program for DVT patients in the ED | • Provider adherence to obtaining the recommended labs  • Provider adherence to evaluating each point in the admission criteria |
| **Angoulvant, 2013 [61]** | Parent therapeutic education on antibiotics | • Attitudes about antibiotic use  • Parent satisfaction about information received in the PED about fever control  • Outcome of the acute infection |
| **Cesarz, 2013 [62]** | Emergency department discharge prescription interventions by emergency medicine pharmacists | • Care providers' satisfaction |

**References:**

1. Hoffmann TC, Glasziou PP, Boutron I, et al. Better reporting of interventions: template for intervention description and replication (TIDieR) checklist and guide. BMJ. 2014;348:g1687. https://doi.org/10.1136/bmj.g1687
2. Kaucher KA, Acquisto NM, Gilliam E, et al. Improvement in HIV screening follow-up rates with emergency medicine pharmacist counseling and dispensing of postexposure prophylaxis for sexual assault patients. J Am Pharm Assoc (Pract Innov). 2025;2(1):100025. https://doi.org/10.1016/j.japhpi.2024.100025
3. Boot A, Weideling A, Wilson A, et al. Impact of a pharmacy-driven culture callback protocol on antimicrobial therapy optimization in the emergency department. J Am Pharm Assoc (2003). 2024;64(4):102072. https://doi.org/10.1016/j.japh.2024.102072
4. Reilly C, Buikstra E, Strivens E, et al. Multidisciplinary care of older adults in the emergency department to influence deprescribing in older adults: a cohort study. J Pharm Pract Res. 2024;54(2):125–134. https://doi.org/10.1002/jppr.1896
5. Lee ES, Louey S, Bushby N, et al. Reducing medication errors on emergency department discharge: evaluation of a collaborative pharmacist-medical officer discharge prescription planning model in a tertiary hospital emergency short stay unit. Emerg Med Australas. 2024;36(4):563–570. https://doi.org/10.1111/1742-6723.14400
6. Selman K, Roberts E, Niznik J, et al. Initiative to deprescribe high-risk drugs for older adults presenting to the emergency department after falls. J Am Geriatr Soc. 2024;72 Suppl 3:S60–S67. https://doi.org/10.1111/jgs.18947
7. Martínez MF, Herrada L, Gutiérrez-Cáceres C, et al. Effect of a clinical pharmacist on 30-day revisits following discharge from an emergency department: a randomized controlled clinical trial. Emergencias. 2024;36(1):33–40. https://doi.org/10.55633/s3me/02.2023
8. Maleki S, Gu G, Buntine P, et al. The effect of an extended-hours ED clinical pharmacy service on admission medication prescribing errors. Emerg Med Australas. 2024 ;36(5):688–694. https://doi.org/10.1111/1742-6723.14415
9. Sofeso ST, Plasencia E, Safri AA, et al. Pharmacist review of discharge antibiotics for urinary tract infections in the emergency department. J Am Coll Clin Pharm. 2024;7(10):996–1003. https://doi.org/10.1002/jac5.2009
10. Tran-Nguyen S, Asha SE. A collaborative pharmacist-led intervention to prevent hospital readmissions among elderly patients discharged from the emergency department: a retrospective cohort study. Sci Rep. 2024;14:15285. https://doi.org/10.1038/s41598-024-64968-8
11. Atey TM, Peterson GM, Salahudeen MS, et al. Redesigning medication management in the emergency department: the impact of partnered pharmacist medication charting on the time to administer pre-admission time-critical medicines, medication order completeness, and venous thromboembolism risk assessment. Pharmacy (Basel). 2024;12(2):71. https://doi.org/10.3390/pharmacy12020071
12. Wang Y, Knobloch K, Lovett S, et al. A pharmacist-driven deprescribing protocol for negative urine and sexually transmitted infection cultures in the emergency department increases antibiotic-free days. Am J Health Syst Pharm. 2024;81(3):e83–e89. https://doi.org/10.1093/ajhp/zxad255
13. Kofoed B, Morris J, Rich J. An initiative to stop antibiotics prescribed for urinary symptoms when urine culture is negative. J Healthc Qual. 2023;45(6):371–376. https://doi.org/10.1097/jhq.0000000000000403
14. Pham D, Lee S, Abrishami S, et al. Utilization and impact of pharmacist-led, urinary culture follow-up after discharge from the emergency department. West J Emerg Med. 2023;24(3):396–400. https://doi.org/10.5811/westjem.59116
15. Jovevski JJ, Smith CR, Roberts JL, et al. Implementation of a compulsory clinical pharmacist-led medication deprescribing intervention in high-risk seniors in the emergency department. Acad Emerg Med. 2023;30(4):410–419. https://doi.org/10.1111/acem.14699
16. Andrade J, Truong J, Ciaramella C. Expansion of a pharmacist-led culture follow-up program to real-time notification of multidrug-resistant microbiology results in the emergency department. Hosp Pharm. 2023;58(4):368–375. https://doi.org/10.1177/00185787231155833
17. Dunn TE, Desai KJ, Krajewski MP, et al. Pharmacists and transitions of care from emergency department to home. Am J Manag Care. 2023;29(12):715–719. https://doi.org/10.37765/ajmc.2023.89473
18. Atey TM, Peterson GM, Salahudeen MS, et al. The impact of partnered pharmacist medication charting in the emergency department on the use of potentially inappropriate medications in older people. Front Pharmacol. 2023 Nov 7;14:1273655. https://doi.org/10.3389/fphar.2023.1273655
19. Atey TM, Peterson GM, Salahudeen MS, et al. Clinical and economic impact of partnered pharmacist medication charting in the emergency department. Front Pharmacol. 2023 Dec 8;14:1273657. https://doi.org/10.3389/fphar.2023.1273657
20. Atey TM, Peterson GM, Salahudeen MS, et al. Impact of partnered pharmacist medication charting (PPMC) on medication discrepancies and errors: a pragmatic evaluation of an emergency department-based process redesign. Int J Environ Res Public Health. 2023;20(2):1452. https://doi.org/10.3390/ijerph20021452
21. Zhao Y, Stornelli N, McAllister K, et al. Evaluating the impact of emergency department clinical pharmacist integration within a discharge urine culture follow-up program. J Am Coll Clin Pharm. 2023;6(9):1008–1014. https://doi.org/10.1002/jac5.1853
22. Benson M, Dewey M, Friesner D. Pharmacist-led urine culture follow-ups in a rural emergency department. J Am Pharm Assoc (2003). 2023;63(4S):S39–S42.e1. https://doi.org/10.1016/j.japh.2022.08.019
23. Ng C, Kowalski S, Mu W, et al. Evaluating smoking cessation service at an emergency department clinical observation unit. Am J Manag Care. 2022;28(10):e388-e391. https://doi.org/10.37765/ajmc.2022.89256
24. Nymoen LD, Flatebø TE, Moger TA, et al. Impact of systematic medication review in emergency department on patients’ post-discharge outcomes—a randomized controlled clinical trial. PLoS One. 2022;17(9):e0274907. https://doi.org/10.1371/journal.pone.0274907
25. Ogilvie M, Nissen L, Kyle G, et al. An evaluation of a collaborative pharmacist prescribing model compared to the usual medical prescribing model in the emergency department. Res Social Adm Pharm. 2022;18(10):3744–3750. https://doi.org/10.1016/j.sapharm.2022.05.005
26. Stewart B, Brody A, Garwood CL, et al. Implementation of outpatient pharmacist-led hypertension management for under-resourced patients: a pilot study. Innov Pharm. 2021;12(2). https://doi.org/10.24926/iip.v12i2.3895
27. Lineberry E, Rozycki E, Jordan TA, et al. Implementation of pharmacist targeted discharge prescription review in an emergency department. Am J Emerg Med. 2021;48:288–294. https://doi.org/10.1016/j.ajem.2021.04.054
28. Celikkayalar E, Puustinen J, Palmgren J, et al. Collaborative medication reviews to identify inappropriate prescribing in pre-admission medications at emergency department short-term ward. Integr Pharm Res Pract. 2021;10:23–32. https://doi.org/10.2147/iprp.s280523
29. Castillo J, Campbell MJ, Sokn E, et al. Discharge prescription optimization by emergency medicine pharmacists in an academic emergency department in the United States. Int J Clin Pharm. 2021;43(3):673–680. https://doi.org/10.1007/s11096-020-01179-x
30. Rainess RA, Patel VV, Cavanaugh JB, et al. Evaluating the addition of a clinical pharmacist service to a midlevel provider-driven culture follow-up program in a community emergency department. J Pharm Technol. 2021;37(3):140–146. https://doi.org/10.1177/87551225211000363
31. Olson A, Feih J, Feldman R, et al. Involvement of pharmacist-reviewed urine cultures and sexually transmitted infections in the emergency department reduces time to antimicrobial optimization. Am J Health Syst Pharm. 2020;77(Suppl 2):S54–S58. https://doi.org/10.1093/ajhp/zxaa064
32. Roels C, Pavich E, McCrate B, et al. Implementing a pharmacist-led emergency department microbiology follow-up collaborative practice agreement. J Am Pharm Assoc (2003). 2020;60(5):e64–e69. https://doi.org/10.1016/j.japh.2020.03.007
33. Lim HY, Lambros P, Krishnamoorthi B, et al. Outpatient management of deep vein thrombosis using direct oral anticoagulants is safe and efficient. J Pharm Pract Res. 2020;50(4):351–355. https://doi.org/10.1002/jppr.1645
34. Tweedle J, Mercado E, Truesdale N, et al. Decreasing review and notification times of genital and urine cultures in a pediatric emergency department: An observational before and after study. JACEP Open. 2020;1(6):1512–1519. https://doi.org/10.1002/emp2.12189
35. Wu JY, Balmat R, Kahle ML, et al. Evaluation of a health system-wide pharmacist-driven emergency department laboratory follow-up and antimicrobial management program. Am J Emerg Med. 2020;38(12):2591–2595. https://doi.org/10.1016/j.ajem.2019.12.052
36. Houlind MB, Andersen AL, Treldal C, et al. A collaborative medication review including deprescribing for older patients in an emergency department: A longitudinal feasibility study. J Clin Med. 2020;9(2):348. https://doi.org/10.3390/jcm9020348
37. Layman SN, Elliott WV, Regen SM, et al. Implementation of a pharmacist-led transitional care clinic. Am J Health Syst Pharm. 2020;77(12):966–971. https://doi.org/10.1093/ajhp/zxaa080
38. Pearson SM, Tandon A, Fixen DR, et al. Pharmacist-led transition of care pilot targeting older people after emergency department discharge. Sr Care Pharm. 2020;35(6):273–282. https://doi.org/10.4140/tcp.n.2020.273
39. Kitchen SA, McGrail K, Wickham ME, et al. Emergency department-based medication review on outpatient health services utilization: interrupted time series. BMC Health Serv Res. 2020;20(1):1–10. https://doi.org/10.1186/s12913-020-05108-6
40. Shealy SC, Alexander C, Hardison TG, et al. Pharmacist-driven culture and sexually transmitted infection testing follow-up program in the emergency department. Pharmacy. 2020;8(2):72. https://doi.org/10.3390/pharmacy8020072
41. Loborec SM, Bazan JA, Brown NV, et al. Privileging pharmacists improves time to patient notification in the microbiological test review process for patients discharged from the emergency department. Am J Health Syst Pharm. 2020;77(Suppl 1):S19–S24. https://doi.org/10.1093/ajhp/zxz335
42. Eswaran V, Allen KC, Cruz DS, et al. Development of a take-home naloxone program at an urban academic emergency department. J Am Pharm Assoc (2003). 2020;60(6):e324–331. https://doi.org/10.1016/j.japh.2020.06.017
43. Giruzzi ME, Tawwater JC, Grelle JL. Evaluation of antibiotic utilization in an emergency department after implementation of an antimicrobial stewardship pharmacist culture review service. Hosp Pharm. 2020;55(4):261–267. https://doi.org/10.1177/0018578719844171
44. Cabilan CJ, Boyde M, Currey E. The effectiveness of pharmacist-led discharge medication counselling in the emergency department (ExPLAIN): a pilot quasi-experimental study. Patient Educ Couns. 2019 Jun 1;102(6):1157–1163. https://doi.org/10.1016/j.pec.2019.01.020
45. Schwab K, Smith R, Wager E, et al. Identification and early anticoagulation in patients with atrial fibrillation in the emergency department. Am J Emerg Med. 2021;44:315–322. https://doi.org/10.1016/j.ajem.2020.04.019
46. Fay LN, Wolf LM, Brandt KL, et al. Pharmacist-led antimicrobial stewardship program in an urgent care setting. Am J Health Syst Pharm. 2019;76(3):175–181. https://doi.org/10.1093/ajhp/zxy023
47. Southerland LT, Porter BL, Newman NW, et al. The feasibility of an inter-professional transitions of care service in an older adult population. Am J Emerg Med. 2019;37(3):553–556. https://doi.org/10.1016/j.ajem.2018.07.047
48. Lacy MC, Bryant GA, Herring MS, et al. Implementation and evaluation of a pharmacy-driven transitions of care program for patients discharged from the emergency department. J Am Coll Clin Pharm. 2019;2(1):8–13. https://doi.org/10.1002/jac5.1011
49. Santolaya-Perrín R, Calderón-Hernanz B, Jiménez-Díaz G, et al. The efficacy of a medication review programme conducted in an emergency department. Int J Clin Pharm. 2019;41(3):757–766. https://doi.org/10.1007/s11096-019-00836-0
50. Chu A, Limberg J. Rivaroxaban program for acute venous thromboembolism upon ED discharge, with focus on utility of commercially available dose pack. Am J Emerg Med. 2017;35(12):1910–1914. https://doi.org/10.1016/j.ajem.2017.08.001
51. DiRenzo BM, Beam DM, Kline JA, et al. Implementation and preliminary clinical outcomes of a pharmacist-managed venous thromboembolism clinic for patients treated with rivaroxaban post emergency department discharge. Acad Emerg Med. 2018;25(6):634–640. https://doi.org/10.1111/acem.13311
52. Hohl CM, Partovi N, Ghement I, et al. Impact of early in-hospital medication review by clinical pharmacists on health services utilization. PLoS One. 2017;12(2):e0170495. https://doi.org/10.1371/journal.pone.0170495
53. Zdyb EG, Courtney DM, Malik S, et al. Impact of discharge anticoagulation education by emergency department pharmacists at a tertiary academic medical center. J Emerg Med. 2017;53(6):896–903. https://doi.org/10.1016/j.jemermed.2017.06.008
54. Hohner E, Ortmann M, Murtaza U, et al. Implementation of an emergency department-based clinical pharmacist transitions-of-care program. Am J Health Syst Pharm. 2016;73(15):1180–1187. https://doi.org/10.2146/ajhp150511
55. Lingenfelter E, Drapkin Z, Fritz K, et al. ED pharmacist monitoring of provider antibiotic selection aids appropriate treatment for outpatient UTI. Am J Emerg Med. 2016;34(8):1600–1603. https://doi.org/10.1016/j.ajem.2016.05.076
56. Santolaya-Perrín R, Jiménez-Díaz G, Galán-Ramos N, et al. A randomised controlled trial on the efficacy of a multidisciplinary health care team on morbidity and mortality of elderly patients attending the Emergency Department. Study design and preliminary results. Farm Hosp. 2016;40(5):371–384. https://doi.org/10.7399/fh.2016.40.5.10465
57. Okere AN, Renier CM, Tomsche JJ. Evaluation of the influence of a pharmacist-led patient-centered medication therapy management and reconciliation service in collaboration with emergency department physicians. J Manag Care Spec Pharm. 2015;21(4):298–306. https://doi.org/10.18553/jmcp.2015.21.4.298.
58. Briggs S, Pearce R, Dilworth S, et al. Clinical pharmacist review: a randomised controlled trial. Emerg Med Australas. 2015;27(5):419–426. https://doi.org/10.1111/1742-6723.12451
59. Falconieri L, Thomson L, Oettinger G, et al. Facilitating anticoagulation for safer transitions: preliminary outcomes from an emergency department deep vein thrombosis discharge program. Hosp Pract (1995). 2014;42(4):16–45. https://doi.org/10.3810/hp.2014.10.1140
60. Dumkow LE, Kenney RM, MacDonald NC, et al. Impact of a multidisciplinary culture follow-up program of antimicrobial therapy in the emergency department. Infect Dis Ther. 2014;3(1):45–53. https://doi.org/10.1007/s40121-014-0026-x
61. Angoulvant F, Rouault A, Prot-Labarthe S, et al. Randomized controlled trial of parent therapeutic education on antibiotics to improve parent satisfaction and attitudes in a pediatric emergency department. PLoS One. 2013;8(9):e75590. https://doi.org/10.1371/journal.pone.0075590
62. Cesarz JL, Steffenhagen AL, Svenson J, et al. Emergency department discharge prescription interventions by emergency medicine pharmacists. Ann Emerg Med. 2013 Feb;61(2):209-214.e1. https://doi.org/10.1016/j.annemergmed.2012.04.011
63. Davis KA, Miyares MA, Price-Goodnow VS. Optimizing transition of care through the facilitation of a pharmacist-managed deep vein thrombosis treatment program. J Pharm Pract. 2013;26(4):438–441. https://doi.org/10.1177/0897190012465953
64. Randolph TC, Parker A, Meyer L, et al. Effect of a pharmacist-managed culture review process on antimicrobial therapy in an emergency department. Am J Health Syst Pharm. 2011;68(10):916–919. https://doi.org/10.2146/ajhp090552
65. Mortimer C, Emmerton L, Lum E. The impact of an aged care pharmacist in a department of emergency medicine. J Eval Clin Pract. 2011;17(3):478–485. https://doi.org/10.1111/j.1365-2753.2010.01454.x
